# The Application of Gamification in Children’s Oral Health Management: Systematic Review

**DOI:** 10.2196/75541

**Published:** 2025-11-04

**Authors:** Jinsong Chen, Ying Ying, Mingli Pang, Jiahao Chen, Ting Kang, Ping Xuan, Xuepeng Chen, Weifang Zhang

**Affiliations:** 1 School of Law Hangzhou City University Hangzhou China; 2 School of Public Affairs Zhejiang University Hangzhou China; 3 School of Population Health University of Auckland Auckland New Zealand; 4 Yongkang Women and Children’s Health Hospital Yongkang China; 5 Stomatology Hospital, School of Stomatology, Zhejiang University School of Medicine, Clinical Research Center for Oral Diseases of Zhejiang Province, Key Laboratory of Oral Biomedical Research of Zhejiang Province, Cancer Center of Zhejiang University Zhejiang University Hangzhou China

**Keywords:** gamification, oral health, digital health, children, pediatric oral health management

## Abstract

**Background:**

Oral health is essential for children’s overall well-being, yet dental caries remain a significant global health issue. Gamification has gained attention as an innovative approach to improving children’s oral health by enhancing engagement and promoting behavior change. Although previous studies have examined different aspects of gamification in pediatric oral health, no review has addressed it as a comprehensive framework encompassing education, behavior change, engagement, and digital platforms.

**Objective:**

This systematic review aimed to evaluate the effectiveness of gamification in pediatric oral health management, focusing on game design elements; intervention platforms; and outcomes such as oral hygiene knowledge, behavior, and engagement.

**Methods:**

Following the PRISMA (Preferred Reporting Items for Systematic Reviews and Meta-Analyses) guidelines, a comprehensive search was conducted across multiple databases (PubMed, MEDLINE, Embase, Cochrane Library, Scopus, Web of Science, and PsycINFO), identifying studies from January 1, 2014, to February 9, 2025. The review included randomized controlled trials, quasi-experimental studies, cohort studies, systematic reviews, and qualitative studies with measurable outcomes that focused on gamified interventions for children’s oral health. A standardized form was used to collect study details, interventions, demographics, outcomes, and risk of bias. Data synthesis was conducted using a narrative approach due to the heterogeneity of the included studies. The synthesis focused on identifying common themes, evaluating intervention effectiveness, and highlighting methodological strengths and limitations.

**Results:**

In total, 41 studies were reviewed, and most (n=24, 59%) used digital interventions such as mobile apps, serious games, and augmented reality. Game elements such as rewards, progress tracking, and interactive feedback were commonly used to enhance user engagement and improve health outcomes. The effect of the digital-based interventions was generally positive, with 38% (9/24) of the studies reporting improvements in oral health knowledge and behaviors. In nonexperimental studies, gamified interventions demonstrated substantial improvements in parental engagement and awareness of oral health practices, which translated into better brushing habits and increased preventive dental visits. Digital-based interventions were more effective in fostering long-term behavior change compared to nondigital alternatives. Engagement metrics revealed higher participation rates in gamified interventions, with improved adherence to oral hygiene routines. However, most studies (39/41, 95%) exhibited moderate risk of bias, including self-reported data and potential selection biases.

**Conclusions:**

This review synthesized findings from 41 studies on gamification in children’s oral health. Gamification, particularly mobile apps and augmented reality, has potential to significantly enhance pediatric oral health management by increasing engagement, improving oral hygiene behaviors, and promoting sustained health behavior change. Future research should explore the long-term effects of these interventions, develop culturally adaptive tools, and integrate gamification with existing public health programs to maximize their impact.

**Trial Registration:**

PROSPERO CRD42025644118; https://www.crd.york.ac.uk/PROSPERO/view/CRD42025644118

## Introduction

### Background

Oral health is a fundamental component of overall well-being [1], particularly in children. It influences their growth, nutrition, speech development, and self-esteem [2,3]. Despite advancements in dental care, childhood dental caries remain a prevalent global health issue, affecting millions of children worldwide [4]. The rising burden of childhood oral health problems highlights the need for engaging strategies to promote early oral hygiene.

Gamification, the application of game design elements in nongame contexts [5,6], has emerged as an innovative approach to health education, leveraging intrinsic motivation and engagement to encourage behavior change [7-9]. In pediatric oral health management, gamification techniques have been integrated into various digital and nondigital interventions, including mobile apps, interactive video games, augmented reality (AR), and game-based learning tools [10,11].

Several studies have demonstrated the effectiveness of gamified oral health education [8,12,13]. A study found that game-based teaching significantly improved children’s oral hygiene knowledge and behaviors compared to conventional methods [10]. Similarly, another study also reported that gamified interventions such as quizzes and crosswords led to higher engagement and better oral hygiene scores among children compared to traditional educational methods [14]. Moreover, digital interventions such as serious games and apps promote sustained behavior change through real-time feedback and personalized reinforcement [11,15].

Despite evidence supporting gamification in children’s oral health management [16-18], challenges remain in optimizing its design, accessibility, and long-term impact. Future research should develop culturally adaptive and age-appropriate gamified interventions, assess their long-term effectiveness, and integrate them with public health programs.

### Rationale

While previous studies have explored various aspects of gamification in pediatric oral health [10,14], no single review has comprehensively examined gamification as a holistic framework for oral health management, including its role in education, behavior change, engagement, and digital platforms.

A preliminary screening of existing systematic reviews was conducted by the study team via the PROSPERO database and identified 4 potentially relevant reviews. The first one, conducted by Elkin et al [19], assessed the effectiveness of oral hygiene tools but did not specifically address gamification. The second one, authored by Rajeh and Mutairi [20], examined the effectiveness of gamification in improving oral health knowledge, practices, and attitudes among school-aged children; this study is somewhat related to ours but is limited to knowledge, practices, and attitudes, possibly excluding broader aspects of gamification applications. The third review, conducted by Patil and Bhandi [21], focused exclusively on game-based teaching methods for children’s oral health. This review specifically examined game-based teaching methods but appears narrower in scope than our study. Finally, Patil et al [22] systematically analyzed pediatric dentistry mobile apps but did not concentrate on gamification strategies for oral health management. Our review explored gamification as a comprehensive strategy encompassing education, behavioral engagement, and digital interventions. It addresses gaps in the literature by evaluating the effectiveness, challenges, and potential improvements of gamification in children’s oral health management.

### Objectives

This review aimed to evaluate gamification’s application and effectiveness in children’s oral health management by addressing the following research questions: (1) how has gamification been implemented in pediatric oral health management, particularly in the context of education and behavior change? (2) what are the key game design elements used in oral health gamification interventions? (3) how effective are gamified interventions in improving children’s oral health knowledge, behaviors, engagement, and education?

## Methods

### Protocol and Registration

This systematic review followed the PRISMA (Preferred Reporting Items for Systematic Reviews and Meta-Analyses) guidelines [23]. It was registered with PROSPERO under registration CRD42025644118.

### Eligibility Criteria

The eligibility criteria are outlined in Textbox 1.

Inclusion criteria were children and adolescents (aged ≤18 years [24]); interventions using gamification strategies for oral health management; and measurable outcomes related to knowledge, behavior, and adherence to oral hygiene practices. Eligible studies included randomized controlled trials (RCTs), quasi-experimental studies, cohort studies, and systematic reviews. While qualitative studies with measurable outcomes were initially considered for inclusion, the systematic search did not identify any that met the eligibility criteria. Only English-language publications were included.

Exclusion criteria were studies focusing on adults; studies that were not available for download and review; interventions lacking gamification elements; qualitative studies without measurable outcomes, such as development studies; and non–English-language publications.

To ensure comprehensiveness, this review also included relevant systematic, scoping, and other types of reviews. These reviews helped capture both research-based and commercially available apps or games, some of which were not included in previous research.

**Eligibility criteria.**

**Inclusion criteria**

- Population: children and adolescents (aged ≤18 years)
- Intervention: gamification strategies for oral health management
- Comparison: any comparison group
- Outcome: related to knowledge, behavior, and adherence to oral hygiene practices
- Study design: randomized controlled trials, quasi-experimental studies, cohort studies, systematic reviews, and qualitative studies with measurable outcomes
- Language: English-language publications

**Exclusion criteria**

- Population: studies focusing on adults (aged >18 years)
- Intervention: interventions lacking gamification elements
- Comparison: none
- Outcome: other outcomes
- Study design: qualitative studies without measurable outcomes (intervention development studies)
- Language: non–English-language publications

### Information Sources

Seven databases were searched. Multimedia Appendix 1 shows the rationale for inclusion.

### Search Strategy

The search strategy targeted 3 main concepts: gamification, pediatric populations, and oral health. Boolean operators (AND and OR) were used to refine search queries. Searches were conducted in PubMed, MEDLINE, Embase, Cochrane Library, Scopus, Web of Science, and PsycINFO from January 1, 2014, to February 9, 2025. Detailed information can be found in Multimedia Appendix 2.

### Study Selection

The study selection process followed the PRISMA guidelines. Screening was conducted in 3 sequential rounds. First, duplicate records were identified and removed. Second, titles and abstracts were screened based on predefined inclusion and exclusion criteria. Finally, full-text articles were assessed for availability and relevance to the review objectives. Studies that met all eligibility criteria were included in the final synthesis. Screening was conducted independently by 2 reviewers (Jinsong Chen and YY), with discrepancies resolved through discussion or consultation with a third reviewer (WZ). Detailed information was illustrated in a PRISMA flowchart.

### Data Extraction

Data extraction was conducted using a structured approach based on multiple established guidelines, including the Transparent Reporting of Evaluations With Nonrandomized Designs statement [25], PRISMA guidelines [23], CONSORT (Consolidated Standards of Reporting Trials) 2010 statement [26], and Strengthening the Reporting of Observational Studies in Epidemiology (STROBE) statement [27]. Additional tools were used to evaluate specific aspects, such as the Mobile App Rating Scale for app assessment [28] and the Risk of Bias in Nonrandomized Studies of Interventions (ROBINS-I) [29] and revised Cochrane risk-of-bias tool for randomized trials (RoB 2) [30] for risk-of-bias evaluation.

The reviewers used EndNote (Clarivate Analytics) for reference management and duplicate removal, followed by Microsoft Excel for study screening and data extraction. A standardized form was used to collect study details, as well as information on interventions, demographics, outcomes, and risk of bias. Two reviewers (Jinsong Chen and YY) extracted the data independently, resolving discrepancies through discussion or a third reviewer (WFZ). Multimedia Appendix 3 presents the extraction table.

### Risk-of-Bias Assessment

Multiple established tools were used to ensure a comprehensive evaluation. The ROBINS-I tool was applied to assess nonrandomized studies, whereas the RoB 2 tool was used for RCTs [29,30]. In addition, the PRISMA guidelines were followed to enhance the transparency and reliability of the assessment [23]. Other relevant tools, including the STROBE statement for observational studies, were also used [27]. Two reviewers (Jinsong Chen and YY) independently evaluated each study, resolving discrepancies through discussion or a third reviewer (WFZ).

### Data Synthesis

A narrative synthesis approach was used. Due to the heterogeneity in study designs and variations in outcome measures across the studies, a meta-analysis was not feasible. In addition, subgroup analyses could not be conducted as different RCTs used distinct outcome measures, making direct comparisons impractical. The synthesis focused on identifying common themes, evaluating intervention effectiveness, and highlighting methodological strengths and limitations.

## Results

### Study Selection

**Figure 1:**
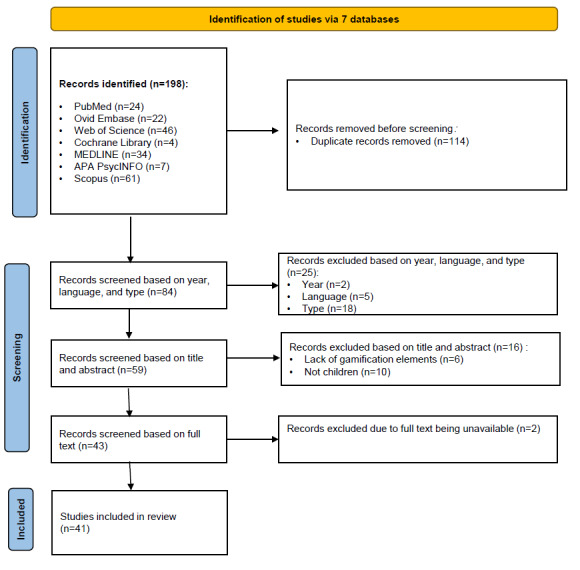
PRISMA (Preferred Reporting Items for Systematic Reviews and Meta-Analyses) flowchart illustrating the study selection process.

The study selection process followed the PRISMA guidelines (Figure 1), resulting in 41 studies.

### Study Characteristics

#### Overview

The reviewed studies were divided into experimental and nonexperimental studies. In total, 59% (24/41) of the studies were experimental, including RCTs, usability studies, and cohort studies. A total of 41% (17/41) of the studies were nonexperimental, encompassing systematic reviews, app evaluations, and development studies. These studies often focused on app functionality, user engagement, and technological innovations [31].

#### Characteristics of the Experimental Studies

The experimental studies were predominantly RCTs, which are considered the gold standard for evaluating intervention effectiveness [7,10,12,14,32-39]. Other designs included usability studies [40-42] and cohort studies [39,43]. These studies were primarily conducted in school-based settings [7,10,14,32-35,44], followed by specialized clinics [12,36,44,45] and home-based environments [12,33]. The population size ranged from small (N=30) to larger groups (N≥500), with a predominant focus on children aged 3 to 15 years [7,10,32,35]. Common interventions evaluated included digital-based games [7,10,12,14,32-38] and mobile health apps [12,32,34,45]. Outcome measures often assessed oral hygiene knowledge, plaque index scores, and behavior changes [10,12,14,32,34]. Multimedia Appendix 4 [4,7,10,12,14,18,32-41,43-50] shows the characteristics of the reviewed experimental studies.

#### Characteristics of the Nonexperimental Studies

The nonexperimental studies encompassed a range of designs, including systematic reviews, app evaluations, and research and development studies. Systematic reviews evaluated interventions such as mobile health apps and serious games [11,51-55]. The app evaluations reviewed included mobile apps targeting children’s oral health, with studies assessing usability, engagement, and effectiveness [17,31,56-58]. A key intervention was the use of mobile health apps and serious games targeting children and parents [51,57,59,60]. These studies commonly evaluated outcomes such as oral health knowledge, behavior changes, plaque index, and engagement levels [16,51,56]. In addition, the studies highlighted a growing focus on assessing the effectiveness of digital interventions in diverse settings, such as schools, dental clinics, and mobile app user testing environments [11,54]. Multimedia Appendix 5 [11,16,17,31,51-63] shows the characteristics of the reviewed nonexperimental studies.

### Risk of Bias

RCTs were evaluated using the Cochrane RoB 2 tool [7,33], whereas nonrandomized and quasi-experimental studies were assessed using the ROBINS-I tool [43,46]. Systematic and scoping reviews adhered to the PRISMA guidelines [11,55], and observational studies followed the STROBE recommendations [58].

RCTs often faced attrition bias and measurement [12], nonrandomized studies exhibited selection bias and confounding [61], and systematic reviews were susceptible to selection and publication biases [52].

Most studies (39/41, 95%) had a moderate risk of bias, mainly due to self-reported measures, intervention adherence variations, and methodological differences. Some RCTs (10/13, 77%) exhibited low risk in domains such as randomization and blinding [37]; others had inconsistencies in measurement and reporting, affecting reliability. Multimedia Appendix 6 [4,7,10-12,14,16-18,31-41,43-63] shows a summary of the risk of bias.

### Synthesis of Results

#### Interventions Discussed in the Experimental Studies

The reviewed experimental studies included various gamified interventions, with 54% (13/24) using digital platforms such as tablet-based games [7,32] and mobile apps [12,34,37,38,42]. Mobile apps were the most common platform (9/13, 69%). These mobile apps used popular technologies such as Android-based apps (5/9, 56%) and AR (2/9, 22%) [33,34]. Gamified elements, including rewards, progress tracking, and real-time feedback, were featured in 29% (7/24) of the studies [12,34,42]. Theoretical frameworks included social cognitive theory (3/24, 12%), behavioral reinforcement (3/24, 12%), and the Behavior Change Wheel (1/24, 4%) [7,32,42]. Multimedia Appendix 7 [4,7,10,12,14,18,32-41,43-50] shows details of all the discussed interventions in the reviewed experimental studies.

#### Interventions Discussed in the Nonexperimental Studies

The nonexperimental studies included various gamified interventions, with 76% (13/17) focusing on digital-based ones. These interventions primarily used mobile apps (9/13, 69%), with platforms such as Android and iOS being the most commonly used [11,52,56,57]. Other technologies included web-based platforms (1/13, 8%), smart toothbrush integration (4/13, 31%), and tablet-based games (2/13, 15%) [17,51]. Rewards, progress tracking, interactive feedback, and real-time quizzes were commonly used (9/17, 53% of the studies) [51,57,59]. Theoretical frameworks were diverse, with the theory of planned behavior and behavior change strategies being prominent [53,59,60]. Multimedia Appendix 8 [11,16,17,31,51-63] shows the interventions discussed in the nonexperimental studies.

#### Narrative Summary of Key Findings in the Experimental Studies

Multimedia Appendix 9 [4,7,10,12,14,18,32-41,43-50] shows the summary of key findings of the reviewed experimental studies. The reviewed studies showed significant improvements in gamified intervention’s effectiveness in oral-health related self-management, behavior, and engagement. A total of 54% (13/24) of the studies used digital-based interventions, which consistently outperformed nondigital alternatives in improving oral health outcomes. For instance, children using gamified mobile health apps [12,34] exhibited superior plaque control compared to those using simple apps. In addition, the use of AR-assisted toothbrushes resulted in a significant reduction in bacterial count when compared to manual toothbrushing (*P*<.05) [34].

Behavioral impacts were notably higher in digital interventions incorporating interactive elements. One study demonstrated a significant improvement in oral hygiene (*P*<.001) in the gamified group, indicating the effectiveness of rewards and visual aids [46]. Similarly, another study observed enhanced brushing independence and skill retention after a gesture-based motion capture game (*P*<.001), highlighting the positive impact of interactive gaming [41].

Engagement metrics also showed that digital interventions with gamified elements fostered sustained involvement. In one study, the regular use rate was significantly higher (72.4%) in the gamified mobile health app group than in the nongamified intervention group (48.3%) [12]. These higher engagement levels were attributed to the use of interactive features such as quizzes, virtual rewards, and progress tracking [42].

Theoretical frameworks, including the behavior change wheel [42] and social cognitive theory [7], were essential in guiding the interventions to ensure alignment with behavior change goals, thereby improving both effectiveness and engagement metrics. These frameworks also helped create interventions that facilitated long-term behavior change, as evidenced by the significant improvements in brushing habits and plaque control [39].

#### Narrative Summary of Key Findings in the Nonexperimental Studies

The nonexperimental studies highlighted the positive impact of gamified interventions on effectiveness in oral-health related self-management, behavior, and engagement in oral health. Digital interventions often incorporated gamified elements such as progress tracking, rewards, and interactive learning modules, contributing to improved adherence to oral hygiene routines [51,59]. For instance, 58.8% of caregivers reported that their children brushed more often when using gamified applications [45].

Regarding behavioral impacts, mobile apps incorporating gamification techniques could increase awareness of preventive dental measures, improve brushing habits, and influence dietary choices [57,60]. Interactive feedback and rewards motivated children to engage more regularly with the interventions. Notably, children exposed to games such as card-based oral health education [62] or mobile self-examination apps [57] showed heightened enthusiasm for learning and improved compliance with brushing techniques.

Engagement metrics were also closely tied to the use of game elements, particularly in applications incorporating quizzes, progress tracking, and real-time feedback. A study that used quiz-based challenges in mobile apps encouraged higher participation and retention than traditional educational methods [61]. Studies using digital rewards and leaderboards reported increased motivation and long-term engagement [16,53].

Theoretical frameworks such as behavioral reinforcement [56] and the theory of planned behavior [60] were essential in developing interventions. These frameworks contributed to shaping effective game mechanics that promoted behavior change and sustained engagement. Multimedia Appendix 10 [11,16,17,31,51-63] summarizes the key findings from the reviewed nonexperimental studies.

## Discussion

### Principal Findings

This synthesis of findings from experimental and nonexperimental studies reveals important trends in the development, evaluation, and effectiveness of gamified interventions in oral health management. The reviewed studies covered a range of intervention types, from serious games [51,56] to mobile health apps [12,59] and AR tools [34]. A notable characteristic of these interventions was their reliance on game elements such as rewards, progress tracking, and interactive learning to enhance user engagement and improve health outcomes.

The effectiveness of digital-based interventions was generally positive, with 38% (9/24) of the experimental studies reporting improvements in oral health knowledge and behaviors [12,59]. For instance, in the gamified app group in one study, plaque control was superior (*P*<.05) compared to the conventional method group [12]. These interventions also showed improved engagement metrics, with higher participation rates especially when game elements such as badges and rewards were incorporated [45,61]. For example, 72.4% of children in the gamified app group in one study engaged regularly compared to 48.3% in the nongamified intervention group [12].

In nonexperimental studies, gamified interventions such as mobile health apps [57,60] demonstrated significant improvements in parental engagement and awareness of oral health practices, which translated into better brushing habits and increased preventive dental visits. The game elements used, such as quizzes and interactive feedback, were particularly effective in driving sustained engagement and behavior changes [51].

However, most studies (39/41, 95%) exhibited moderate risk of bias, including self-reported data and potential selection biases [4,56]. The theoretical frameworks used, such as behavioral reinforcement and the theory of planned behavior, were consistent with improvements in both engagement and effectiveness [44,60]. Despite the variability in study designs, gamified interventions consistently demonstrated promise in improving oral health outcomes, especially when complemented with engaging, interactive features.

### Interpretation

The studies in this review explored the effectiveness of gamified interventions in oral health education and management. The experimental studies consistently showed that gamified interventions significantly improved oral health behaviors and engagement [10,12]. For example, the gamified mobile health app group in one study showed superior plaque control (*P*<.05) compared to the nongamified control group, and higher engagement rates were observed in interventions incorporating rewards and progress tracking [12]. These results align with those of previous research that emphasizes the positive impact of digital gamification in health interventions [11,52], which underscores the role of gamification in improving health knowledge and motivation, especially among children.

However, the nonexperimental studies further expanded the understanding of the role of gamification, particularly with regard to long-term engagement and user satisfaction. For instance, mobile health apps [57,60] showed improvements in parental engagement with educational content, which led to better oral health practices and dietary choices. These studies point to the growing importance of gamification in motivating not only children but also parents in managing oral health [59]. This supports the current research, which highlights gamified apps as particularly effective in both enhancing knowledge and encouraging behavior change.

While both experimental and nonexperimental studies showed promising results, the limitations of many of the reviewed studies, such as lack of controlled trials and small sample sizes, remain an issue, which is consistent with findings from previous reviews [11,56].

### Limitations and Recommendations

First, there was significant heterogeneity across the studies in terms of intervention types, outcome measures, and populations, making direct comparisons challenging [60]. Different gamified elements were used across the studies, making it difficult to pinpoint which specific features were most effective [16]. In addition, many studies (22/41, 54%) relied on self-reported data, which introduced potential biases related to participant recall and response [12,44]. Furthermore, while the PROSPERO registration proposed including qualitative studies, the final synthesis was limited to quantitative designs as the systematic search did not identify any qualitative studies that met the eligibility criteria. Finally, several studies (10/41, 24%) lacked long-term follow-up as most focused on short-term outcomes [32,33]. These limitations highlight the need for future studies to adopt more standardized designs, integrate educational frameworks into gamification strategies, and include multistakeholder collaboration to ensure that gamified children oral health tools are practically effective. Long-term follow-up and mixed methods approaches are also recommended to provide a more comprehensive understanding of user experiences and effectiveness.

### Conclusions

This systematic review synthesized the findings from 41 studies on the use of gamification in children’s oral health management. Gamified interventions, particularly those incorporating digital technologies such as mobile apps and AR, showed significant improvements in children’s oral health knowledge, brushing behaviors, and plaque control. The effectiveness of these interventions was notably enhanced when gamification elements such as rewards, progress tracking, and interactive feedback were used. In addition, the theoretical frameworks used, including behavior change models and game-based learning principles, played a crucial role in fostering engagement and improving health outcomes.

## Data Availability

All data relevant to this study are included in this paper or uploaded as supplementary information.

## Appendix

Multimedia Appendix 1 Selected databases and rationale for selection.

Multimedia Appendix 2 Search terms entered into different databases.

Multimedia Appendix 3 Formatted data extraction table.

Multimedia Appendix 4 Summary of characteristics of the reviewed experimental studies.

Multimedia Appendix 5 Summary of characteristics of the reviewed nonexperimental studies.

Multimedia Appendix 6 Summary of risk-of-bias findings of the reviewed studies.

Multimedia Appendix 7 Interventions discussed in the experimental studies.

Multimedia Appendix 8 Interventions discussed in the nonexperimental studies.

Multimedia Appendix 9 Summary of key findings of the reviewed experimental studies.

Multimedia Appendix 10 Summary of key findings of the reviewed nonexperimental studies.

Multimedia Appendix 11 PRISMA checklist.

## Abbreviations

AR: augmented reality
CONSORT: Consolidated Standards of Reporting Trials
PRISMA: Preferred Reporting Items for Systematic Reviews and Meta-Analyses
RCT: randomized controlled trial
RoB 2: revised Cochrane risk-of-bias tool for randomized trials
ROBINS-I: Risk of Bias in Nonrandomized Studies of Interventions
STROBE: Strengthening the Reporting of Observational Studies in Epidemiology
