# Supplementary material for: The Application of Gamification in Children’s Oral Health Management: Systematic Review
Source: J Med Internet Res. 2025 Nov 4;27:e75541. doi: 10.2196/75541 (PMC12627974; doi:10.2196/75541)
Supplement: Multimedia Appendix 1 [file jmir_v27i1e75541_app1.docx]

## Appendix 1: Selected Databases and Rationales of Selection

| **Databases (n=7)** | **Rationales** |
| --- | --- |
| PubMed | Covers biomedical and life sciences literature, including oral health, gamification, and digital health interventions. |
| MEDLINE (via Ovid) | Provides structured indexing using MeSH* terms for efficient retrieval of oral health and gamification studies. |
| Embase (via Ovid) | Strong in European and pharmaceutical research, valuable for digital health interventions and health technology. |
| Cochrane Library | Premier source for systematic reviews and RCTs, offering high-quality evidence on gamification and oral health interventions. |
| Scopus | Multidisciplinary database with extensive peer-reviewed journal coverage, relevant to gamification in healthcare and education. |
| Web of Science | Facilitates citation tracking and interdisciplinary research, ensuring inclusion of behavioral science and pediatric oral health studies. |
| PsycINFO (via Ovid) | Focuses on psychology and behavioral sciences, useful for studies on behavior change, motivation, and engagement in oral health interventions. |

* MeSH: Medical Subject Headings
