# Supplementary material for: The Application of Gamification in Children’s Oral Health Management: Systematic Review
Source: J Med Internet Res. 2025 Nov 4;27:e75541. doi: 10.2196/75541 (PMC12627974; doi:10.2196/75541)
Supplement: Multimedia Appendix 2 [file jmir_v27i1e75541_app2.docx]

## Appendix 2: Search Terms Entered in Different Databases

| **Databases** | **Search term 1** | **Search term 2** | **Search term 3** | **Search strategy** |
| --- | --- | --- | --- | --- |
| PubMed | ("Gamification"[MeSH] OR "Serious Games"[tiab:~0] OR "Game-Based Learning"[tiab:~0] OR "Educational Games"[tiab:~0] OR gamif* OR "game-based" OR "serious game*" OR "interactive game*" OR "digital game*" OR "exergam*" OR "game mechanic*") | ("Child"[MeSH] OR "Adolescent"[MeSH] OR "Pediatrics"[MeSH] OR child* OR adolescen* OR pediatric* OR youth OR teen* OR school* OR "young people") | ("Oral Health"[MeSH] OR "Dental Health"[tiab:~0] OR "Tooth Diseases"[MeSH] OR "Dental Caries"[MeSH] OR "Preventive Dentistry"[MeSH] OR "Oral Hygiene"[MeSH] OR "Pediatric Dentistry"[MeSH] OR "Tooth Brushing"[tiab:~0] OR "dental care" OR "oral care" OR "oral health behav*" OR "teeth cleaning" OR "floss*" OR "brushing behav*") | #1 AND #2 AND #3 |
| MEDLINE (via Ovid) | (Gamification/ or "Serious Games"/ or "Game-Based Learning"/ or "Educational Games"/ or gamif*.mp. or "game-based".mp. or "serious game*".mp. or "interactive game*".mp. or "digital game*".mp. or "exergam*".mp. or "game mechanic*".mp.) | (Child Development/ or Pediatric Psychology/ or Adolescents/ or Children/ or child*.mp. or adolescen*.mp. or pediatric*.mp. or youth.mp. or teen*.mp. or school*.mp. or "young people".mp.) | (Oral Hygiene/ or "Dental Health"/ or "Preventive Dentistry"/ or "Toothbrushing"/ or "Pediatric Dentistry"/ or "oral health".mp. or "dental care".mp. or "oral hygiene".mp. or "tooth brushing".mp. or "oral health behav*".mp. or "teeth cleaning".mp. or "floss*".mp. or "brushing behav*".mp.) | #1 AND #2 AND #3 |
| Embase (via Ovid) | (('gamification' or 'game-based learning' or 'serious games').mp. or gamif*.ti,ab. or 'game-based'.ti,ab. or 'digital game*'.ti,ab.) | ('child' or 'pediatric' or child* or adolescen* or pediatric* or youth or teen* or school*).mp. | ('oral health' or 'dental care' or 'oral hygiene' or 'tooth brushing' or 'pediatric dentistry' or 'oral health behav*').mp. | #1 AND #2 AND #3 |
| Cochrane Library | gamif* OR “game-based” OR (serious NEXT game*) OR (interactive NEXT game*) OR (digital NEXT game*) OR exergam* OR (game NEXT mechanic*) OR (educational NEXT game*)  [All Text] | child* OR adolescen* OR pediatric* OR youth OR teen* OR school* OR “young people”  [All Text] | “oral health” OR “dental care” OR “oral hygiene” OR “tooth brushing” OR “pediatric dentistry” OR (oral NEXT health NEXT behav*) OR “teeth cleaning” OR floss* OR (brushing NEXT behav*)  [All Text] | #1 AND #2 AND #3 |
| Scopus | (gamif* OR "game-based" OR "serious game*" OR "interactive game*" OR "digital game*" OR "exergam*" OR "game mechanic*" OR "Game-Based Learning" OR "Educational Games")  [TITLE-ABS-KEY] | (child* OR adolescen* OR pediatric* OR youth OR teen* OR school* OR "young people")  [TITLE-ABS-KEY] | ("oral health" OR "dental care" OR "oral hygiene" OR "tooth brushing" OR "pediatric dentistry" OR "oral health behav*" OR "teeth cleaning" OR floss* OR "brushing behav*")  [TITLE-ABS-KEY] | #1 AND #2 AND #3 |
| Web of Science | (gamif* OR "game-based" OR "serious game*" OR "interactive game*" OR "digital game*" OR "exergam*" OR "game mechanic*" OR "Game-Based Learning" OR "Educational Games")  [All Fields] | (child* OR adolescen* OR pediatric* OR youth OR teen* OR school* OR "young people")  [All Fields] | ("oral health" OR "dental care" OR "oral hygiene" OR "tooth brushing" OR "pediatric dentistry" OR "oral health behav*" OR "teeth cleaning" OR floss* OR "brushing behav*")  [All Fields] | #1 AND #2 AND #3 |
| PsycINFO | (Gamification/ or "Serious Games"/ or "Game-Based Learning"/ or "Educational Games"/ or gamif*.mp. or "game-based".mp. or "serious game*".mp. or "interactive game*".mp. or "digital game*".mp. or "exergam*".mp. or "game mechanic*".mp.) | (Child Development/ or Pediatric Psychology/ or Adolescents/ or Children/ or child*.mp. or adolescen*.mp. or pediatric*.mp. or youth.mp. or teen*.mp. or school*.mp. or "young people".mp.) | (Oral Hygiene/ or "Dental Health"/ or "Preventive Dentistry"/ or "Toothbrushing"/ or "Pediatric Dentistry"/ or "oral health".mp. or "dental care".mp. or "oral hygiene".mp. or "tooth brushing".mp. or "oral health behav*".mp. or "teeth cleaning".mp. or "floss*".mp. or "brushing behav*".mp.) | #1 AND #2 AND #3 |
