# Supplementary material for: The Application of Gamification in Children’s Oral Health Management: Systematic Review
Source: J Med Internet Res. 2025 Nov 4;27:e75541. doi: 10.2196/75541 (PMC12627974; doi:10.2196/75541)
Supplement: Multimedia Appendix 3 [file jmir_v27i1e75541_app3.docx]

## Appendix 3: Formatted Data Extraction Table

| **Section** | **Q#** | **Data Extracted** | **Details** |
| --- | --- | --- | --- |
| Study Identification |  | Reference Number | Unique identifier (e.g., 2, 3, 4, … 45) |
|  | 1 | Author(s) & Year | Full citation (First author, year) |
|  | 2 | Title | Full title of the study |
|  | 3 | Journal/Source | Name of the journal or conference |
| Study Design | 4 | Study Type | RCT, Quasi-Experimental, Systematic Review, Digital Tool Development, Observational Study |
|  | 5 | Research Objective | Main aim of the study |
|  | 6 | Sample Size | Number of participants (if applicable) |
|  | 7 | Study Setting | Location (e.g., hospital, school, online) |
| Intervention Characteristics | 8 | Type of Gamified Intervention | Game-based learning, digital game, serious game, chatbot, etc. |
|  | 9 | Platform/Technology Used | Mobile app, VR, AR, wearables, board game, interactive tool |
|  | 10 | Game Elements Used | Points, rewards, challenges, leaderboards, personalization |
|  | 11 | Theoretical Framework | Behavior change model (e.g., COM-B, Self-Determination Theory) |
| Population Characteristics | 12 | Target Population | Children, special needs, adolescents, caregivers |
|  | 13 | Age Group | Mean age or range |
|  | 14 | Inclusion/Exclusion Criteria | Key eligibility conditions |
| Outcomes Measured | 15 | Primary Outcome(s) | Knowledge gain, adherence, plaque reduction, behavior change |
|  | 16 | Secondary Outcome(s) | Engagement, usability, motivation, satisfaction |
|  | 17 | Effectiveness Results | Summary of key findings |
|  | 18 | Behavioral Impact | Changes in brushing, flossing, dental visits |
|  | 19 | Engagement Metrics | User retention, duration of use, gamification effectiveness |
| Study Quality & Risk of Bias Assessment | 20 | Risk of Bias Tool Used | ROBINS-I for non-randomized studies, Cochrane RoB 2 for RCTs |
|  | 21 | Risk of Bias Domains | (1) Confounding, (2) Selection bias, (3) Intervention classification, (4) Deviations from intended interventions, (5) Missing data, (6) Measurement bias, (7) Reporting bias |
|  | 22 | Overall Risk of Bias Rating | Low, Moderate, Serious, Critical |
|  | 23 | Study Limitations | As reported by authors |
| Adaptation & Generalization | 24 | Cultural or Demographic Adaptation | Language, design modifications |
|  | 25 | Technical Adaptation | Platform compatibility, accessibility |
| Conclusion & Relevance | 26 | Study Conclusion | Main conclusion drawn by authors |
|  | 27 | Relevance to Gamified Oral Health | How applicable is this to children’s dental health? |
|  | 28 | Recommendations for Future Research | Suggested improvements or next steps |
