# Supplementary material for: The Application of Gamification in Children’s Oral Health Management: Systematic Review
Source: J Med Internet Res. 2025 Nov 4;27:e75541. doi: 10.2196/75541 (PMC12627974; doi:10.2196/75541)
Supplement: Multimedia Appendix 4 [file jmir_v27i1e75541_app4.docx]

## Appendix 4: Summary of Study Characteristics of Reviewed Experimental Studies

| # | Reference | Study Design and Population | Population and Setting | Interventions | Outcome Measures |
| --- | --- | --- | --- | --- | --- |
| 1 | Panic et al., 2014 | Experimental Study | 190 children (7–9 years old)  Primary schools in Belgium | Compared weak vs. strong threat messages and different media (computer game, brochure, narrative story) to promote dental hygiene. | Adaptive behaviour (healthy snack choice), engagement metrics (self-reported attention). |
| 2 | Aljafari et al., 2015 | RCT | 110 cchildren (4–10 years old)  King’s College Hospital, London | Compared an oral health education video game to verbal oral health education to improve dietary knowledge and oral hygiene habits. | Parent and child satisfaction, dietary knowledge, changes in diet and oral hygiene habits, engagement with the game. |
| 3 | Kumar et al., 2015 | RCT | 60 children (7–10 years old)  School-based, Tamil Nadu, India | Compared conventional (flash card) and game-based (“Connect the Dots”) teaching on oral hygiene knowledge and behaviour. | Oral hygiene knowledge, Debris Index-Simplified (DI-S) score, long-term retention of knowledge, proportion of children with good/fair/poor oral hygiene. |
| 4 | Malik et al., 2017 | RCT | 150 children (8–12 years old)  School-based, Lucknow, India | Compared game-based (crosswords and quizzes) vs. conventional (PowerPoint presentations) oral health education. | Oral health knowledge score, plaque index score, long-term retention of knowledge. |
| 8 | Chuko et al., 2020 | Design and usability study | 204 children (3–6 years old)  Not specified | Developed and evaluated an interactive toothbrush aid with sensory feedback and gamification to improve brushing habits. | Average brushing duration, brushing accuracy, parental feedback on usability and engagement. |
| 10 | Sharififard et al., 2020 | Cluster RCT | 200 children (6–17 years old), visually impaired  Specialized schools, Tehran, Iran | Compared oral health education using Audio Tactile Performance (ATP) technique alone, ATP combined with oral health education for mothers, and ATP with an art-based package on the oral health status of visually impaired children. | Simplified Oral Hygiene Index (OHI-S), Bleeding on Probing (BOP) percentage, comparison of different educational approaches. |
| 11 | Effendi et al., 2021 | Quasi-experimental study | 54 children (7–8 years old)  Elementary school, Malang, Indonesia | Evaluated the effectiveness of reminder sticker books to increase dental health knowledge and improve oral hygiene. | Dental health knowledge, Simplified Oral Hygiene Index (OHI-S), frequency of sticker use. |
| 12 | Kang et al., 2021 | Single-subject experimental design | 4 children (7–11 years old) with intellectual disabilities  Special education classrooms, Taiwan | Evaluated the effectiveness of a Kinect-based interactive video game in teaching oral hygiene skills. | Percentage of independently completed brushing steps, skill retention, parental and teacher assessment of skill improvement, engagement levels. |
| 13 | Sharma et al., 2021 | Prospective cohort study | 300 children (7–12 years old)  Elementary schools, Bareilly, Uttar Pradesh, India | Compared conventional oral health education, game-based education (snakes and ladders), and self-made storybooks in improving oral hygiene. | Oral Hygiene Index-Simplified (OHI-S) Debris (DI-S) score, learning retention. |
| 14 | Shruti et al., 2021 | Non-randomized experimental pre–post study | 220 children (3–6 years old)  Preschools, India | Evaluated the effectiveness of storytelling using hand puppets in improving oral health knowledge, attitudes, and practices. | Mean KAP scores, specific knowledge, attitude, and behaviour components, parental monitoring of habits, engagement levels. |
| 16 | Zolfaghari et al., 2021 | RCT | 51 mother-child pairs (children ≤6 years old)  Not specified | Evaluated a gamified mobile health (mHealth) app designed to improve mothers’ oral health knowledge and practice and its impact on their children's oral hygiene, compared to a simple app. | Oral health knowledge and practice of mothers, dental plaque index (PI) of children, engagement levels. |
| 17 | Aljafari et al., 2022 | RCT | 278 children (6–8 years old)  Primary schools, Amman, Jordan | Evaluated the impact of a video-game-based oral health intervention on children’s dietary knowledge, dietary and toothbrushing practices, plaque scores, and parental familiarity with preventive treatments. | Dietary knowledge, dietary and toothbrushing practices, plaque score, parental familiarity with fluoride varnish and fissure sealants. |
| 18 | Kumar et al., 2022 | RCT | 100 adolescents (12–15 years old)  Private primary school, Belagavi, Karnataka, India | Evaluated the effect of a novel interactive game-based visual performance (IGVP) technique versus a conventional oral health education (OHE) talk on plaque control, gingival health, and oral hygiene knowledge and practices in adolescents. | Plaque index score, gingival index score, oral hygiene knowledge score, engagement levels measured through Kahoot quiz performance, retention of oral hygiene knowledge. |
| 19 | Kashyap et al., 2022 | Field Trial | 160 children (12 years old)  Private school, Lucknow, India | Compared conventional and game-based oral health education on oral hygiene performance. | Plaque index, gingival index, oral hygiene knowledge. |
| 21 | Dey et al., 2023 | Experimental Non-Randomized Concurrent Parallel Single-Blinded Study | 32 children (6-8 years old)  JSS Dental College and Hospital, Mysore, India | Evaluated the effectiveness of an augmented reality-assisted toothbrush in improving oral hygiene using fluorescence imaging and microbiological analysis of plaque Streptococcus mutans count. | Change in fluorescence scores, plaque Streptococcus mutans count. |
| 24 | Jagadeson et al., 2023 | Interventional Study | 120 children (10-12 years old)  School, Chengalpattu District, Tamil Nadu, India | Assessed the effectiveness of the Dental Jumanji game in improving oral health knowledge when combined with conventional lectures. | Change in oral health knowledge scores. |
| 27 | Saraf et al., 2023 | RCT | 51 children (4-6 years old)  University dental college, Maharashtra, India | Compared the educational game “My Tooth the Happiest” with standard dietary counseling in improving preschool children’s preference for non-cariogenic food items. | Change in dietary preference scores for non-cariogenic food items, retention of dietary preference changes. |
| 28 | Shi et al., 2023 | RCT | 160 children (3-6 years old)  Preschools, China | Compared the “Dental Truth or Dare” board game with traditional didactic instruction in improving oral hygiene knowledge and oral hygiene status. | Oral hygiene knowledge scores, debris index scores. |
| 30 | Chang et al., 2024 | Interventional Study | 30 children (5-12 years old)  Pediatric dental clinic, Taiwan | Developed and evaluated an educational chatbot designed to promote oral self-care. | Usability, likeability, and user experience of the chatbot. |
| 31 | France et al., 2024 | Pilot Study | 17 children (5-12 years old) with Autism Spectrum Disorder  Pediatric dental clinic, USA | Examined caregivers’ and children’s experiences with a smart electric toothbrush designed to improve oral hygiene. | Changes in toothbrushing habits, usability and acceptance of the smart toothbrush, engagement levels. |
| 32 | Karkoutly et al., 2024 | Triple-blinded, two-arm, parallel-group, RCT | 60 children (6-10 years old)  Pediatric Dentistry, Damascus University, Syria | Evaluated the effectiveness of the “Baby Panda Dental Care” mobile game in reducing pain and anxiety in children undergoing primary molars pulpotomy, compared with the traditional Tell-Show-Do (TSD) technique. | Pulse rate, Pictorial Scale for subjective dental anxiety assessment, FLACC Behavioral Pain Scale. |
| 38 | Santhosh et al., 2024 | Single-Blind RCT | 195 adolescents (12-15 years old)  Public school, Belagavi, Karnataka, India | Assessed the effectiveness of Jigsaw Puzzle-assisted Visual Reinforcement (JPVR) technique in improving toothbrushing knowledge, practices, and clinical oral health outcomes compared to conventional oral health education (OHE) and video demonstrations among adolescents. | Oral health knowledge and practices, plaque and gingival index scores. |
| 39 | Shirahmadi et al., 2024 | RCT | 190 elementary school girls (11-12 years old)  Elementary schools, Hamadan, Iran | Assessed the impact of an oral health education program on the oral hygiene behaviors and clinical indicators using a theory-based intervention. | Toothbrushing and flossing frequency, gingival bleeding index, dental plaque levels. |
| 40 | Borrelli et al., 2025 | RCT | 754 parents of underserved children  Pediatric clinics, USA | Evaluated parent-targeted oral health text messaging for underserved children attending pediatric clinics. | Caregiver-reported child brushing frequency, flossing frequency, sugary drink consumption, clinical assessment of caries. |

* Raghavendra, Madhuri, Sujata (RMS) Pictorial Scale (RMS-PS)

Reference:

4. Panic K, Cauberghe V, De Pelsmacker P. Promoting dental hygiene to children: comparing traditional and interactive media following threat appeals. J Health Commun. 2014;19(5):561-76. PMID: 24393019. doi: 10.1080/10810730.2013.821551.

7. Aljafari A, Rice C, Gallagher JE, Hosey MT. An oral health education video game for high caries risk children: Study protocol for a randomized controlled trial. Trials. 2015;16(1). doi: 10.1186/s13063-015-0754-6.

10. Kumar Y, Asokan S, John B, Gopalan T. Effect of Conventional and Game-based Teaching on Oral Health Status of Children: A Randomized Controlled Trial. International journal of clinical pediatric dentistry. 2015;8(2):123-6. doi: <https://dx.doi.org/10.5005/jp-journals-10005-1297>.

12. Zolfaghari M, Shirmohammadi M, Shahhosseini H, Mokhtaran M, Mohebbi SZ. Development and evaluation of a gamified smart phone mobile health application for oral health promotion in early childhood: a randomized controlled trial. BMC Oral Health. 2021;21(1):18. PMID: 33413304. doi: 10.1186/s12903-020-01374-2.

14. Malik A, Sabharwal S, Kumar A, Singh Samant P, Singh A, Kumar Pandey V. Implementation of Game-based Oral Health Education <ovid:i>vs</ovid:i> Conventional Oral Health Education on Children's Oral Health-related Knowledge and Oral Hygiene Status. International journal of clinical pediatric dentistry. 2017;10(3):257-60. doi: <https://dx.doi.org/10.5005/jp-journals-10005-1446>.

19. Chang W-J, Chang P-C, Chang Y-H. The gamification and development of a chatbot to promote oral self-care by adopting behavior change wheel for Taiwanese children. Digit Health. 2024;10:20552076241256750. PMID: 38798886. doi: 10.1177/20552076241256750.

29. Aljafari A, ElKarmi R, Nasser O, Atef Aa, Hosey MT. A Video-Game-Based Oral Health Intervention in Primary Schools-A Randomised Controlled Trial. Dentistry journal. 2022;10(5). doi: <https://dx.doi.org/10.3390/dj10050090>.

30. Kumar KRS, Deshpande AP, Ankola AV, Sankeshwari RM, Jalihal S, Hampiholi V, et al. Effectiveness of a Visual Interactive Game on Oral Hygiene Knowledge, Practices, and Clinical Parameters among Adolescents: A Randomized Controlled Trial. Children-Basel. 2022 Dec;9(12). PMID: WOS:000902292000001. doi: 10.3390/children9121828.

31. Dey S, Deshmukh S, Umamaheshwari S, Dheeraj L, Sinchan HG. Fluorescence-based Evaluation of the Efficacy of Augmented Reality-assisted Toothbrush on Oral Hygiene Practices Among 6–8 Years Old Children. Journal of Advanced Oral Research. 2023;14(2):183-9. doi: 10.1177/23202068231193772.

32. Saraf T, Hegde R, Shah P. Comparison of “My Tooth the Happiest” educational game with standard dietary counseling for preference toward non-cariogenic food items in preschool children: A Randomized control trial. Journal of Indian Society of Pedodontics and Preventive Dentistry. 2023;41(1):35-42. doi: 10.4103/jisppd.jisppd_93_23.

33. Santhosh VN, Shankkari S, Coutinho D, Ankola AV, Sankeshwari RM, Hampiholi V, et al. Effectiveness of a toothbrushing intervention utilizing puzzle-solving game assisted with visual aids among adolescents: A single-blind randomized controlled trial. Przegl Epidemiol. 2024 Dec 10;78(3):318-25. PMID: 39660713. doi: 10.32394/pe/195139.

34. Karkoutly M, Al-Halabi MN, Laflouf M, Bshara N. Effectiveness of a dental simulation game on reducing pain and anxiety during primary molars pulpotomy compared with tell-show-do technique in pediatric patients: a randomized clinical trial. BMC Oral Health. 2024;24(1). doi: 10.1186/s12903-024-04732-6.

35. Shirahmadi S, Bashirian S, Soltanian AR, Karimi-Shahanjarini A, Vahdatinia F. Effectiveness of theory-based educational interventions of promoting oral health among elementary school students. BMC Public Health. 2024 Jan 9;24(1):130. PMID: 38195494. doi: 10.1186/s12889-023-17528-0.

36. Borrelli B, Endrighi R, Heeren T, Adams WG, Gansky SA, Werntz S, et al. Parent-Targeted Oral Health Text Messaging for Underserved Children Attending Pediatric Clinics: A Randomized Clinical Trial. JAMA Netw Open. 2025 Jan 2;8(1):e2452780. PMID: 39745701. doi: 10.1001/jamanetworkopen.2024.52780.

37. Chuko C, Chao FL, Tsai HY. Design of interactive AIDS for children's teeth cleaning habits. Advances in Science, Technology and Engineering Systems. 2020;5(2):494-9. doi: 10.25046/aj050263.

38. Kang YS, Chang YJ, Howell SR. Using a kinect-based game to teach oral hygiene in four elementary students with intellectual disabilities. J Appl Res Intellect Disabil. 2021 Mar;34(2):606-14. PMID: 33258262. doi: 10.1111/jar.12828.

40. Sharma S, Saxena S, Naik SN, Bhandari R, Shukla AK, Gupta P. Comparison between Conventional, Game-based, and Self-made Storybook-based Oral Health Education on Children's Oral Hygiene Status: A Prospective Cohort Study. International journal of clinical pediatric dentistry. 2021;14(2):273-7. doi: <https://dx.doi.org/10.5005/jp-journals-10005-1811>.

41. Sharififard N, Sargeran K, Gholami M, Zayeri F. A music- and game-based oral health education for visually impaired school children; multilevel analysis of a cluster randomized controlled trial. BMC Oral Health. 2020 May 18;20(1):144. PMID: 32423446. doi: 10.1186/s12903-020-01131-5.

42. France K, Urquhart O, Ko E, Gomez J, Ryan M, Hernandez M, et al. A Pilot Study Exploring Caregivers' Experiences Related to the Use of a Smart Toothbrush by Children with Autism Spectrum Disorder. Children (Basel). 2024 Apr 11;11(4). PMID: 38671677. doi: 10.3390/children11040460.

55. Effendi MC, Hartami E, Balbeid M, Hapsari GD. Effectiveness of reminder sticker books at increasing dental health knowledge and oral hygiene. Dental Journal. 2021;54(1):5-10. doi: 10.20473/j.djmkg.v54.i1.p5-10.

Newly added

Shruti T, Govindraju HA, Sriranga J. Incorporation of Storytelling as a Method of Oral Health Education among 3-6-year-old Preschool Children. Int J Clin Pediatr Dent. 2021 May-Jun;14(3):349-352. doi: 10.5005/jp-journals-10005-1946. PMID: 34720505; PMCID: PMC8543987.

Kashyap P, Reddy L, Sinha P, Verma I, Adwani J. Effectiveness of Game-Based Oral Health Education Method on Oral Hygiene Performance of 12-Year-Old Private School Children in Lucknow City: A field trial. Journal of Indian Association of Public Health Dentistry. 2022;20:43.

Jagadeson M, Prasad V, Priyadharshini I, Prasad H, Dharshini D, Sethi M. Effect of Game Based Education in Extension of Oral Health Knowledge among 10 -12 Year Old School Children - An Interventional Study. Journal of Oral Health and Oral Epidemiology. 2024;12(4):164-9.

Shi Y, Wu WZ, Huo A, Wang HH, Lu WB, Jin XH. Effect of Conventional and "Dental Truth or Dare" Board Game on Oral Hygiene Knowledge and Oral Hygiene Status of Preschool Children. Games Health J. 2023 Apr;12(2):125-131. doi: 10.1089/g4h.2022.0059. Epub 2022 Dec 27. PMID: 36577043.
