# Supplementary material for: The Application of Gamification in Children’s Oral Health Management: Systematic Review
Source: J Med Internet Res. 2025 Nov 4;27:e75541. doi: 10.2196/75541 (PMC12627974; doi:10.2196/75541)
Supplement: Multimedia Appendix 5 [file jmir_v27i1e75541_app5.docx]

## Appendix 5: Summary of Study Characteristics of Reviewed Non-experimental Studies

| # | Reference | Study Design | Population | Setting | Intervention | Outcome Measures |
| --- | --- | --- | --- | --- | --- | --- |
| 5 | Reynolds et al., 2019 | Observational study (secondary data analysis) | 60 children with early childhood caries (ECC) | King’s College Hospital, London (Paediatric Dentistry and Day Surgery Units) | Online serious game (“Scott’s Hospital Dental Visit”) to prepare children for dental treatment under general anaesthesia | Compliance with game usage (playtime, missed slides); Family engagement patterns (home vs. ward, replays); Child’s preoperative anxiety |
| 6 | Campos et al., 2019 | Observational study (usability testing) | 43 preschool children | Public school, Fortaleza, Brazil | Mobile application for teaching oral health concepts to preschool-aged children | Usability of the mobile app (effectiveness, efficiency, satisfaction); Engagement levels (task completion rates); Challenges in navigation; Differences in engagement by age group; Need for dietary education |
| 7 | Amantini et al., 2020 | Serious game development protocol | Children in the early school-age phase | University research setting (Bauru School of Dentistry, Brazil) | Augmented reality (AR)-based serious game for interactive oral hygiene training | Toothbrushing technique improvement; Error identification and correction during toothbrushing |
| 9 | Fijačko et al., 2020 | Systematic review and app evaluation | NA | App stores (Google Play, Apple App Store, Windows Phone Store, Amazon) | Mobile apps incorporating gamification to support children’s oral hygiene practices | Identification of gamification features; App quality ratings; Evaluation of behaviour change strategies; Consistency of app content with evidence-based dentistry; Analysis of user engagement metrics |
| 15 | Zaror et al., 2021 | Scoping review | NA | Multiple global settings | Serious games for oral health education and professional training | Effectiveness in knowledge acquisition and skill development; Impact on oral health behaviour and adherence; Participant engagement and satisfaction; Quality assessment of the included studies |
| 20 | Ajay et al., 2023 | Systematic Review | NA | Dental clinics, primary care centres, public preschools, and mobile app user testing environments (Germany, Saudi Arabia, USA) | Mobile health applications targeting parents for the prevention of early childhood caries | Effectiveness in improving oral health knowledge among parents; Impact on oral hygiene practices, plaque index, and gingival index; Changes in parental attitudes and behaviours; Usability and engagement levels; Adherence to recommendations |
| 22 | Fegan & Hutchinson, 2023 | Commentary on a systematic review | NA | Dental clinics, primary care centres, public preschools (Germany, Saudi Arabia, USA) | Mobile health applications with gamification for oral health promotion | Improvement in parental oral health knowledge; Change in plaque and gingival indices in children; Behavioural modifications; Parental engagement; Effectiveness of gamification |
| 23 | Gayatri et al., 2023 | Research and Development Study | 30 elementary school students | Malang, Indonesia | Mobile application for self-examination of dental caries and oral hygiene | Usability of the mobile application; Self-reported caries experience; Self-reported oral hygiene status; Validation of application accuracy; Engagement and motivation levels |
| 25 | Mohammadzadeh et al., 2023 | Systematic Review | NA | Multiple countries (Brazil, UK, USA) | Mobile health applications for children’s oral health improvement | Effectiveness in improving oral hygiene knowledge; Impact on plaque and gingival indices; Parental engagement; Usability and acceptance; Effectiveness of gamification |
| 26 | Rizany et al., 2023 | Literature Review | NA | Elementary schools (India, China) | Card games designed for oral health education | Improvement in oral health knowledge; Changes in Oral Hygiene Index-Simplified (OHI-S) and Debris Index-Simplified (DI-S); Engagement and motivation levels; Retention of oral health knowledge |
| 29 | Widodorini & Salsabila, 2023 | Pre-experimental study | 30 elementary school students | Bekasi, Indonesia | Modified Twister educational game application for oral health education | Changes in oral health behaviour scores; Improved oral hygiene practices; Increased enthusiasm; Higher participation |
| 33 | Mendonça et al., 2024 | Descriptive Study (User-Centered Design) | 15 children and their caregivers | Pediatric dental clinic, Brazil | Brazilian version of an oral health education game with user-centered design modifications | Children’s performance in game tasks; User experience feedback; Engagement and motivation levels; Caregivers’ perceptions of educational value |
| 34 | Meriç, 2024 | Cross-Sectional Study (App Quality Evaluation) | Children | Turkish Google Play Store and Apple Store | Gamified oral hygiene mobile applications for children | Overall MARS scores; Evidence-based content evaluation; Comparison of app store user ratings with MARS scores |
| 35 | Moreira et al., 2024 | Scoping Review | NA | Schools, dental clinics, and home-based digital interventions | Various digital interventions (oral self-care apps, mobile games, interactive educational platforms) | Impact on oral health behaviours; Effectiveness in promoting oral health knowledge and engagement; Comparison with traditional methods; Identification of game design elements and behaviour change strategies |
| 36 | Padmanabhan et al., 2024 | Narrative Review | NA | Homes, schools, and dental clinics | Oral hygiene mobile applications incorporating gamification elements | Impact on brushing frequency and technique; Improvement in oral hygiene knowledge and behaviours; Potential for personalized oral hygiene education; Enhanced engagement |
| 37 | Patil et al., 2024 | Systematic Review of RCTs | NA | Schools and dental clinics (India, Jordan, Iran, UK) | Various game-based educational methods (digital video games, board games, crossword puzzles, interactive storybooks) | Improvement in oral health knowledge; Changes in oral hygiene behaviours; Enhanced engagement; Effective learning experiences |
| 41 | Peerbhay et al., 2025 | Scoping Review | NA | Schools, communities, and dental clinics (low- and middle-income populations) | Behavior changes interventions for improving oral health behaviors | Changes in oral hygiene behaviours; Reduction in plaque and caries incidence; Effectiveness of digital and non-digital interventions; Improved oral health knowledge; Engagement levels; Sustainability of behavior change interventions |

Reference:

11. Fijačko N, Gosak L, Cilar L, Novšak A, Creber RM, Skok P, et al. The Effects of Gamification and Oral Self-Care on Oral Hygiene in Children: Systematic Search in App Stores and Evaluation of Apps. JMIR Mhealth Uhealth. 2020;8(7):e16365. PMID: 32673235. doi: 10.2196/16365.

18. Mendonça TS, Carvalho STd, Aljafari A, Hosey MT, Costa LR. Oral Health Education for Children: Development of a Serious Game with a User-Centered Design Approach. Games Health J. 2024;13(4):268-77. PMID: 38563685. doi: 10.1089/g4h.2023.0055.

28. Campos LFXA, Cavalcante JP, Machado DP, Marçal E, Silva PGDB, Rolim JPML. Development and Evaluation of a Mobile Oral Health Application for Preschoolers. Telemedicine and e-Health. 2019;25(6):492-8. doi: 10.1089/tmj.2018.0034.

43. Zaror C, Mariño R, Atala-Acevedo C. Current State of Serious Games in Dentistry: A Scoping Review. Games Health J. 2021;10(2). PMID: 33818135. doi: 10.1089/g4h.2020.0042.

44. Mohammadzadeh N, Gholamzadeh M, Zahednamazi S, Ayyoubzadeh SM. Mobile health applications for children's oral health improvement: A systematic review. Informatics in Medicine Unlocked. 2023 2023/01/01/;37:101189. doi: <https://doi.org/10.1016/j.imu.2023.101189>.

45. Padmanabhan V, D’Souza S, Priya SP, Rehman M, El Bahra S, Tawfiq N, et al. Harnessing the Potential of Oral Hygiene Apps for Pediatric Dental Care: A Comprehensive Narrative Review. Journal of International Dental and Medical Research. 2024;17(2):860-5.

46. Patil S, Licari FW, Bhandi S, Awan KH, Di Blasio M, Isola G, et al. Effect of game-based teaching on the oral health of children: a systematic review of randomised control trials. J Clin Pediatr Dent. 2024 Jul;48(4):26-37. PMID: 39087211. doi: 10.22514/jocpd.2024.075.

47. Peerbhay F, Mash R, Khan S. Effectiveness of oral health promotion in children and adolescents through behaviour change interventions: A scoping review. PLoS One. 2025;20(1):e0316702. PMID: 39792864. doi: 10.1371/journal.pone.0316702.

48. Reynolds PA, Donaldson AN, Liossi C, Newton JT, Donaldson NK, Arias R, et al. How families prepare their children for tooth extraction under general anaesthesia: Family and clinical predictors of non-compliance with a ‘serious game’. International Journal of Paediatric Dentistry. 2019;29(2):117-28. doi: 10.1111/ipd.12450.

49. Gayatri RW, Alma LR, Ashar M, Mohd Nor NA. Smart oral health: A mobile application for dental caries and oral hygiene self-examination. Asia-Pacific Journal of Public Health. 2023;35(8):552-4. doi: <https://dx.doi.org/10.1177/10105395231204987>.

51. Meriç E. Evaluation of the quality of oral hygiene mobile apps for children using the mobile app rating scale. Int J Med Inform. 2024 Dec;192:105612. PMID: 39236585. doi: 10.1016/j.ijmedinf.2024.105612.

52. Ajay K, Azevedo LB, Haste A, Morris AJ, Giles E, Gopu BP, et al. App-based oral health promotion interventions on modifiable risk factors associated with early childhood caries: A systematic review. Frontiers in oral health. 2023;4:1125070. doi: <https://dx.doi.org/10.3389/froh.2023.1125070>.

53. Fegan H, Hutchinson R. Is the answer to reducing early childhood caries in your pocket? Evid Based Dent. 2023 Sep;24(3):134-5. PMID: 37582973. doi: 10.1038/s41432-023-00922-3.

54. Moreira R, Silveira A, Sequeira T, Durao N, Lourenco J, Cascais I, et al. Gamification and Oral Health in Children and Adolescents: Scoping Review. Interactive journal of medical research. 2024;13:e35132. doi: <https://dx.doi.org/10.2196/35132>.

56. Widodorini T, Salsabila AN. The Use of the Modified Twister Educational Game Application as Dental and Oral Health Education Media. Malaysian Journal of Medicine and Health Sciences. 2023;19:28-32. doi: 10.47836/mjmhs.19.3.5.

57. Rizany AK, Christabella J, Sulijaya B. Implementation of Card Games as Educational Media for Dental and Oral Health in Elementary School Children: A Literature Review. Journal of International Dental and Medical Research. 2023;16(3):1323-6.

New added

Amantini SNSR, Montilha AAP, Antonelli BC, Leite KTM, Rios D, Cruvinel T, Lourenço Neto N, Oliveira TM, Machado MAAM. Using Augmented Reality to Motivate Oral Hygiene Practice in Children: Protocol for the Development of a Serious Game. JMIR Res Protoc. 2020 Jan 17;9(1):e10987. doi: 10.2196/10987. PMID: 31951216; PMCID: PMC6996757.
