# Supplementary material for: The Application of Gamification in Children’s Oral Health Management: Systematic Review
Source: J Med Internet Res. 2025 Nov 4;27:e75541. doi: 10.2196/75541 (PMC12627974; doi:10.2196/75541)
Supplement: Multimedia Appendix 6 [file jmir_v27i1e75541_app6.docx]

## Appendix 6: Summary of Risk of Bias Findings of Reviewed Studies

| Study# | Reference | Study Design | Risk of Bias Tool Used | Risk of Bias Domains | Overall Risk of Bias Rating |
| --- | --- | --- | --- | --- | --- |
| 1 | Panic et al, 2014 | Experimental Study (2 × 3 factorial design) | ROBINS-I | Confounding, Selection bias, Intervention classification, Deviations from intended interventions, Missing data, Measurement bias, Reporting bias | Moderate (due to self-reported outcomes and lack of prior knowledge assessment) |
| 2 | Aljafari et al, 2015 | RCT | Cochrane RoB2 | Randomization, Blinding, Attrition Bias, Measurement Bias, Intervention Adherence, Reporting Bias | Moderate (due to self-reported data and potential attrition) |
| 3 | Kumar et al, 2015 | RCT | Cochrane RoB2 | Randomization, Blinding, Attrition Bias, Measurement Bias, Intervention Fidelity, Reporting Bias | Moderate (due to self-reported knowledge scores and lack of long-term follow-up beyond 3 months) |
| 4 | Malik et al, 2017 | RCT | Cochrane RoB2 | Randomization, Blinding, Attrition Bias, Measurement Bias, Intervention Fidelity, Reporting Bias | Moderate (due to self-reported knowledge scores and potential examiner bias) |
| 5 | Reynolds et al, 2019 | Observational study | ROBINS-I | Confounding, Selection Bias, Measurement Bias, Reporting Bias | Moderate (due to reliance on self-reported anxiety and automated logs without observational verification) |
| 6 | Campos et al, 2019 | Observational study | ROBINS-I | Confounding, Selection Bias, Measurement Bias, Reporting Bias | Moderate (due to reliance on usability testing and lack of long-term behavioral impact assessment) |
| 7 | Amantini et al, 2020 | Serious game development protocol | Not applicable | Not applicable | Not applicable |
| 8 | Chuko et al, 2020 | Design and usability study | ROBINS-I | Confounding, Selection Bias, Measurement Bias, Reporting Bias | Moderate (due to reliance on self-reported brushing adherence and limited sample size) |
| 9 | Fijacko et al, 2020 | Systematic review and app evaluation | PRISMA | Selection Bias, Information Bias, Publication Bias | Moderate (due to potential selection bias and reliance on app store data) |
| 10 | Sharififard et al, 2020 | Cluster RCT | Cochrane RoB2 | Randomization, Blinding, Attrition Bias, Measurement Bias, Intervention Fidelity, Reporting Bias | Moderate (due to self-reported adherence and short follow-up period) |
| 11 | Effendi et al, 2021 | Quasi-experimental study | ROBINS-I | Confounding, Selection Bias, Measurement Bias, Reporting Bias | Moderate (due to reliance on self-reported brushing adherence and sticker tracking) |
| 12 | Kang et al,2021 | Single-subject experimental design | ROBINS-I | Confounding, Selection Bias, Measurement Bias, Reporting Bias | Moderate (due to small sample size and observational measures) |
| 13 | Sharma et al, 2021 | Prospective cohort study | ROBINS-I | Confounding, Selection Bias, Measurement Bias, Reporting Bias | Moderate (due to self-reported learning retention and limited follow-up) |
| 14 | Shruti et al, 2021 | Non-randomized experimental pre–post study | ROBINS-I | Confounding, Selection Bias, Measurement Bias, Reporting Bias | Moderate (due to self-reported parental data and short follow-up) |
| 15 | Zaror et al, 2021 | Scoping review | PRISMA | Selection Bias, Information Bias, Publication Bias | Moderate (due to lack of methodological rigor in included studies) |
| 16 | Zolfaghari et al, 2021 | RCT | Cochrane RoB2 | Randomization, Blinding, Attrition Bias, Measurement Bias, Intervention Fidelity, Reporting Bias | Moderate (due to self-reported data and potential performance bias) |
| 17 | Aljafari et al, 2022 | RCT | Cochrane RoB2 | Randomization, Blinding, Attrition Bias, Measurement Bias, Intervention Fidelity, Reporting Bias | Moderate (due to self-reported behavior and low home engagement) |
| 18 | Kumar et al, 2022 | RCT | Cochrane RoB2 | Randomization, Blinding, Attrition Bias, Measurement Bias, Intervention Fidelity, Reporting Bias | Moderate due to self-reported outcomes and short follow-up period |
| 19 | Kashyap et al, 2022 | Field Trial | ROBINS-I | Confounding, Selection Bias, Measurement Bias, Reporting Bias | Moderate due to examiner-dependent assessments and short-term follow-up |
| 20 | Ajay et al, 2023 | Systematic Review | PRISMA guidelines | Confounding, Selection Bias, Measurement Bias, Reporting Bias | Moderate due to variability in study methodologies and limited controlled trials |
| 21 | Dey et al, 2023 | Experimental Non-Randomized Concurrent Parallel Single-Blinded Study | ROBINS-I | Confounding, Selection Bias, Measurement Bias, Reporting Bias | Moderate due to the non-randomized study design and potential examiner bias |
| 22 | Fegan & Hutchinson, 2023 | Commentary on a systematic review | PRISMA guidelines | Confounding, Selection Bias, Measurement Bias, Reporting Bias | Moderate due to study design variations and potential self-reporting bias |
| 23 | Gayatri et al, 2023 | Research and Development Study | ROBINS-I | Confounding, Selection Bias, Measurement Bias, Reporting Bias | Moderate due to the small sample size and lack of a comparative control group |
| 24 | Jagadeson et al, 2023 | Interventional Study | ROBINS-I | Confounding, Selection Bias, Measurement Bias, Reporting Bias | Moderate due to reliance on self-reported knowledge assessments and the non-randomized study design |
| 25 | Mohammadzadeh et al, 2023 | Systematic Review | PRISMA guidelines | Confounding, Selection Bias, Measurement Bias, Reporting Bias | Moderate due to differences in study methodologies and reliance on self-reported outcomes |
| 26 | Rizany et al, 2023 | Literature Review | PRISMA guidelines | Confounding, Selection Bias, Measurement Bias, Reporting Bias | Moderate due to differences in study methodologies and reliance on self-reported assessments in some studies |
| 27 | Saraf et al, 2023 | RCT | Cochrane RoB2 | Randomization, Blinding, Attrition Bias, Measurement Bias, Reporting Bias | Moderate due to partial blinding and participant attrition |
| 28 | Shi et al, 2023 | RCT | Cochrane RoB2 | Randomization, Blinding, Attrition Bias, Measurement Bias, Reporting Bias | Moderate due to partial blinding and reliance on self-reported knowledge assessments |
| 29 | Widodorini  & Salsabila, 2023 | Pre-experimental study with one-group pretest-posttest design | ROBINS-I | Confounding, Selection Bias, Measurement Bias, Reporting Bias | Moderate due to the non-randomized design and absence of a comparative control group |
| 30 | Chang et al, 2024 | Interventional Study | ROBINS-I | Confounding, Selection Bias, Measurement Bias, Reporting Bias | Moderate due to non-randomized design and limited sample size |
| 31 | France et al, 2024 | Pilot Study (Pretest–Posttest, Non-Randomized Clinical Study) | ROBINS-I | Confounding, Selection Bias, Measurement Bias, Reporting Bias | Moderate due to non-randomized design and self-reported caregiver assessments |
| 32 | Karkoutly et al, 2024 | Triple-blinded, two-arm, parallel-group, RCT | Cochrane RoB2 | Randomization, Blinding, Attrition Bias, Measurement Bias, Reporting Bias | Low to moderate due to self-reported anxiety measures and short-term follow-up |
| 33 | Mendonça et al, 2024 | Descriptive Study (User-Centered Design Approach) | ROBINS-I | Confounding, Selection Bias, Measurement Bias, Reporting Bias | Moderate due to the small sample size and lack of a control group |
| 34 | Meriç, 2024 | Cross-Sectional Study (App Quality Evaluation) | STROBE guidelines | Selection Bias, Measurement Bias, Reporting Bias | Moderate due to the limited number of apps reviewed and exclusion of paid apps |
| 35 | Moreira et al, 2024 | Scoping Review | PRISMA guidelines | Selection Bias, Measurement Bias, Reporting Bias | Moderate due to heterogeneous study designs and varying sample sizes |
| 36 | Padmanabhan et al, 2024 | Narrative Review | PRISMA guidelines | Selection Bias, Measurement Bias, Reporting Bias | Moderate due to variability in study methodologies |
| 37 | Patil et al, 2024 | Systematic Review | Cochrane RoB2 | Selection Bias, Measurement Bias, Reporting Bias | Moderate due to variability in study methodologies and limited long-term follow-up data |
| 38 | Santhoshet al, 2024 | Single-Blind RCT | Cochrane RoB2 | Randomization, Blinding, Attrition Bias, Measurement Bias, Reporting Bias | Moderate due to reliance on self-reported measures and the short follow-up period |
| 39 | Shirahmadi et al, 2024 | RCT | Cochrane RoB2 | Randomization, Blinding, Attrition Bias, Measurement Bias, Reporting Bias | Moderate due to self-reported data and lack of long-term follow-up |
| 40 | Borrelli et al, 2025 | RCT | Cochrane RoB2 | Randomization, Blinding, Attrition Bias, Measurement Bias, Reporting Bias | Moderate due to reliance on self-reported outcomes and attrition in dental assessments |
| 41 | Peerbhay et al, 2025 | Scoping Review | PRISMA guidelines | Selection Bias, Measurement Bias, Reporting Bias | Moderate due to variability in study designs and quality |

Reference:

4. Panic K, Cauberghe V, De Pelsmacker P. Promoting dental hygiene to children: comparing traditional and interactive media following threat appeals. J Health Commun. 2014;19(5):561-76. PMID: 24393019. doi: 10.1080/10810730.2013.821551.

7. Aljafari A, Rice C, Gallagher JE, Hosey MT. An oral health education video game for high caries risk children: Study protocol for a randomized controlled trial. Trials. 2015;16(1). doi: 10.1186/s13063-015-0754-6.

10. Kumar Y, Asokan S, John B, Gopalan T. Effect of Conventional and Game-based Teaching on Oral Health Status of Children: A Randomized Controlled Trial. International journal of clinical pediatric dentistry. 2015;8(2):123-6. doi: <https://dx.doi.org/10.5005/jp-journals-10005-1297>.

11. Fijačko N, Gosak L, Cilar L, Novšak A, Creber RM, Skok P, et al. The Effects of Gamification and Oral Self-Care on Oral Hygiene in Children: Systematic Search in App Stores and Evaluation of Apps. JMIR Mhealth Uhealth. 2020;8(7):e16365. PMID: 32673235. doi: 10.2196/16365.

12. Zolfaghari M, Shirmohammadi M, Shahhosseini H, Mokhtaran M, Mohebbi SZ. Development and evaluation of a gamified smart phone mobile health application for oral health promotion in early childhood: a randomized controlled trial. BMC Oral Health. 2021;21(1):18. PMID: 33413304. doi: 10.1186/s12903-020-01374-2.

14. Malik A, Sabharwal S, Kumar A, Singh Samant P, Singh A, Kumar Pandey V. Implementation of Game-based Oral Health Education <ovid:i>vs</ovid:i> Conventional Oral Health Education on Children's Oral Health-related Knowledge and Oral Hygiene Status. International journal of clinical pediatric dentistry. 2017;10(3):257-60. doi: <https://dx.doi.org/10.5005/jp-journals-10005-1446>.

18. Mendonça TS, Carvalho STd, Aljafari A, Hosey MT, Costa LR. Oral Health Education for Children: Development of a Serious Game with a User-Centered Design Approach. Games Health J. 2024;13(4):268-77. PMID: 38563685. doi: 10.1089/g4h.2023.0055.

19. Chang W-J, Chang P-C, Chang Y-H. The gamification and development of a chatbot to promote oral self-care by adopting behavior change wheel for Taiwanese children. Digit Health. 2024;10:20552076241256750. PMID: 38798886. doi: 10.1177/20552076241256750.

28. Campos LFXA, Cavalcante JP, Machado DP, Marçal E, Silva PGDB, Rolim JPML. Development and Evaluation of a Mobile Oral Health Application for Preschoolers. Telemedicine and e-Health. 2019;25(6):492-8. doi: 10.1089/tmj.2018.0034.

29. Aljafari A, ElKarmi R, Nasser O, Atef Aa, Hosey MT. A Video-Game-Based Oral Health Intervention in Primary Schools-A Randomised Controlled Trial. Dentistry journal. 2022;10(5). doi: <https://dx.doi.org/10.3390/dj10050090>.

30. Kumar KRS, Deshpande AP, Ankola AV, Sankeshwari RM, Jalihal S, Hampiholi V, et al. Effectiveness of a Visual Interactive Game on Oral Hygiene Knowledge, Practices, and Clinical Parameters among Adolescents: A Randomized Controlled Trial. Children-Basel. 2022 Dec;9(12). PMID: WOS:000902292000001. doi: 10.3390/children9121828.

31. Dey S, Deshmukh S, Umamaheshwari S, Dheeraj L, Sinchan HG. Fluorescence-based Evaluation of the Efficacy of Augmented Reality-assisted Toothbrush on Oral Hygiene Practices Among 6–8 Years Old Children. Journal of Advanced Oral Research. 2023;14(2):183-9. doi: 10.1177/23202068231193772.

32. Saraf T, Hegde R, Shah P. Comparison of “My Tooth the Happiest” educational game with standard dietary counseling for preference toward non-cariogenic food items in preschool children: A Randomized control trial. Journal of Indian Society of Pedodontics and Preventive Dentistry. 2023;41(1):35-42. doi: 10.4103/jisppd.jisppd_93_23.

33. Santhosh VN, Shankkari S, Coutinho D, Ankola AV, Sankeshwari RM, Hampiholi V, et al. Effectiveness of a toothbrushing intervention utilizing puzzle-solving game assisted with visual aids among adolescents: A single-blind randomized controlled trial. Przegl Epidemiol. 2024 Dec 10;78(3):318-25. PMID: 39660713. doi: 10.32394/pe/195139.

34. Karkoutly M, Al-Halabi MN, Laflouf M, Bshara N. Effectiveness of a dental simulation game on reducing pain and anxiety during primary molars pulpotomy compared with tell-show-do technique in pediatric patients: a randomized clinical trial. BMC Oral Health. 2024;24(1). doi: 10.1186/s12903-024-04732-6.

35. Shirahmadi S, Bashirian S, Soltanian AR, Karimi-Shahanjarini A, Vahdatinia F. Effectiveness of theory-based educational interventions of promoting oral health among elementary school students. BMC Public Health. 2024 Jan 9;24(1):130. PMID: 38195494. doi: 10.1186/s12889-023-17528-0.

36. Borrelli B, Endrighi R, Heeren T, Adams WG, Gansky SA, Werntz S, et al. Parent-Targeted Oral Health Text Messaging for Underserved Children Attending Pediatric Clinics: A Randomized Clinical Trial. JAMA Netw Open. 2025 Jan 2;8(1):e2452780. PMID: 39745701. doi: 10.1001/jamanetworkopen.2024.52780.

37. Chuko C, Chao FL, Tsai HY. Design of interactive AIDS for children's teeth cleaning habits. Advances in Science, Technology and Engineering Systems. 2020;5(2):494-9. doi: 10.25046/aj050263.

38. Kang YS, Chang YJ, Howell SR. Using a kinect-based game to teach oral hygiene in four elementary students with intellectual disabilities. J Appl Res Intellect Disabil. 2021 Mar;34(2):606-14. PMID: 33258262. doi: 10.1111/jar.12828.

40. Sharma S, Saxena S, Naik SN, Bhandari R, Shukla AK, Gupta P. Comparison between Conventional, Game-based, and Self-made Storybook-based Oral Health Education on Children's Oral Hygiene Status: A Prospective Cohort Study. International journal of clinical pediatric dentistry. 2021;14(2):273-7. doi: <https://dx.doi.org/10.5005/jp-journals-10005-1811>.

41. Sharififard N, Sargeran K, Gholami M, Zayeri F. A music- and game-based oral health education for visually impaired school children; multilevel analysis of a cluster randomized controlled trial. BMC Oral Health. 2020 May 18;20(1):144. PMID: 32423446. doi: 10.1186/s12903-020-01131-5.

42. France K, Urquhart O, Ko E, Gomez J, Ryan M, Hernandez M, et al. A Pilot Study Exploring Caregivers' Experiences Related to the Use of a Smart Toothbrush by Children with Autism Spectrum Disorder. Children (Basel). 2024 Apr 11;11(4). PMID: 38671677. doi: 10.3390/children11040460.

43. Zaror C, Mariño R, Atala-Acevedo C. Current State of Serious Games in Dentistry: A Scoping Review. Games Health J. 2021;10(2). PMID: 33818135. doi: 10.1089/g4h.2020.0042.

44. Mohammadzadeh N, Gholamzadeh M, Zahednamazi S, Ayyoubzadeh SM. Mobile health applications for children's oral health improvement: A systematic review. Informatics in Medicine Unlocked. 2023 2023/01/01/;37:101189. doi: <https://doi.org/10.1016/j.imu.2023.101189>.

45. Padmanabhan V, D’Souza S, Priya SP, Rehman M, El Bahra S, Tawfiq N, et al. Harnessing the Potential of Oral Hygiene Apps for Pediatric Dental Care: A Comprehensive Narrative Review. Journal of International Dental and Medical Research. 2024;17(2):860-5.

46. Patil S, Licari FW, Bhandi S, Awan KH, Di Blasio M, Isola G, et al. Effect of game-based teaching on the oral health of children: a systematic review of randomised control trials. J Clin Pediatr Dent. 2024 Jul;48(4):26-37. PMID: 39087211. doi: 10.22514/jocpd.2024.075.

47. Peerbhay F, Mash R, Khan S. Effectiveness of oral health promotion in children and adolescents through behaviour change interventions: A scoping review. PLoS One. 2025;20(1):e0316702. PMID: 39792864. doi: 10.1371/journal.pone.0316702.

48. Reynolds PA, Donaldson AN, Liossi C, Newton JT, Donaldson NK, Arias R, et al. How families prepare their children for tooth extraction under general anaesthesia: Family and clinical predictors of non-compliance with a ‘serious game’. International Journal of Paediatric Dentistry. 2019;29(2):117-28. doi: 10.1111/ipd.12450.

49. Gayatri RW, Alma LR, Ashar M, Mohd Nor NA. Smart oral health: A mobile application for dental caries and oral hygiene self-examination. Asia-Pacific Journal of Public Health. 2023;35(8):552-4. doi: <https://dx.doi.org/10.1177/10105395231204987>.

51. Meriç E. Evaluation of the quality of oral hygiene mobile apps for children using the mobile app rating scale. Int J Med Inform. 2024 Dec;192:105612. PMID: 39236585. doi: 10.1016/j.ijmedinf.2024.105612.

52. Ajay K, Azevedo LB, Haste A, Morris AJ, Giles E, Gopu BP, et al. App-based oral health promotion interventions on modifiable risk factors associated with early childhood caries: A systematic review. Frontiers in oral health. 2023;4:1125070. doi: <https://dx.doi.org/10.3389/froh.2023.1125070>.

53. Fegan H, Hutchinson R. Is the answer to reducing early childhood caries in your pocket? Evid Based Dent. 2023 Sep;24(3):134-5. PMID: 37582973. doi: 10.1038/s41432-023-00922-3.

54. Moreira R, Silveira A, Sequeira T, Durao N, Lourenco J, Cascais I, et al. Gamification and Oral Health in Children and Adolescents: Scoping Review. Interactive journal of medical research. 2024;13:e35132. doi: <https://dx.doi.org/10.2196/35132>.

55. Effendi MC, Hartami E, Balbeid M, Hapsari GD. Effectiveness of reminder sticker books at increasing dental health knowledge and oral hygiene. Dental Journal. 2021;54(1):5-10. doi: 10.20473/j.djmkg.v54.i1.p5-10.

56. Widodorini T, Salsabila AN. The Use of the Modified Twister Educational Game Application as Dental and Oral Health Education Media. Malaysian Journal of Medicine and Health Sciences. 2023;19:28-32. doi: 10.47836/mjmhs.19.3.5.

57. Rizany AK, Christabella J, Sulijaya B. Implementation of Card Games as Educational Media for Dental and Oral Health in Elementary School Children: A Literature Review. Journal of International Dental and Medical Research. 2023;16(3):1323-6.

New added:

Shruti T, Govindraju HA, Sriranga J. Incorporation of Storytelling as a Method of Oral Health Education among 3-6-year-old Preschool Children. Int J Clin Pediatr Dent. 2021 May-Jun;14(3):349-352. doi: 10.5005/jp-journals-10005-1946. PMID: 34720505; PMCID: PMC8543987.

Kashyap P, Reddy L, Sinha P, Verma I, Adwani J. Effectiveness of Game-Based Oral Health Education Method on Oral Hygiene Performance of 12-Year-Old Private School Children in Lucknow City: A field trial. Journal of Indian Association of Public Health Dentistry. 2022;20:43.

Jagadeson M, Prasad V, Priyadharshini I, Prasad H, Dharshini D, Sethi M. Effect of Game Based Education in Extension of Oral Health Knowledge among 10 -12 Year Old School Children - An Interventional Study. Journal of Oral Health and Oral Epidemiology. 2024;12(4):164-9.

Shi Y, Wu WZ, Huo A, Wang HH, Lu WB, Jin XH. Effect of Conventional and "Dental Truth or Dare" Board Game on Oral Hygiene Knowledge and Oral Hygiene Status of Preschool Children. Games Health J. 2023 Apr;12(2):125-131. doi: 10.1089/g4h.2022.0059. Epub 2022 Dec 27. PMID: 36577043.

Amantini SNSR, Montilha AAP, Antonelli BC, Leite KTM, Rios D, Cruvinel T, Lourenço Neto N, Oliveira TM, Machado MAAM. Using Augmented Reality to Motivate Oral Hygiene Practice in Children: Protocol for the Development of a Serious Game. JMIR Res Protoc. 2020 Jan 17;9(1):e10987. doi: 10.2196/10987. PMID: 31951216; PMCID: PMC6996757.
