# Supplementary material for: The Application of Gamification in Children’s Oral Health Management: Systematic Review
Source: J Med Internet Res. 2025 Nov 4;27:e75541. doi: 10.2196/75541 (PMC12627974; doi:10.2196/75541)
Supplement: Multimedia Appendix 7 [file jmir_v27i1e75541_app7.docx]

## Appendix 7: Interventions discussed in Experimental Studies

| # | Reference | Type of Gamified Intervention | Platform/Technology Used | Digital-Based Intervention | Game Elements Used | Theoretical Framework |
| --- | --- | --- | --- | --- | --- | --- |
| 1 | Panic et al., 2014 | Educational computer game | Computer-based interactive game | Yes | Narrative, interactive tasks, decision-making | Extended Parallel Processing Model (EPPM) |
| 2 | Aljafari et al., 2015 | Educational video game | Tablet-based game (iPad) with DVD home use option | Yes | Interactive avatar, decision-making, storytelling | Social Cognitive Theory (SCT) |
| 3 | Kumar et al., 2015 | "Connect the Dots" game with flash cards | Paper-based game intervention | No | Sequential learning, visual reinforcement | Cognitive Learning Theory (Piaget’s Concrete Operational Stage) |
| 4 | Malik et al., 2017 | Crossword and quiz-based learning | Paper-based games integrated with PowerPoint presentations | No | Puzzles, quizzes, interactive questions | Cognitive Learning Theory |
| 8 | Chuko et al., 2020 | Smart toothbrush with LED guide | Arduino-based microcontroller, Bluetooth connectivity, inertial sensors, LED feedback | Yes | Brushing position tracking, interactive lighting, reward system | Behavioral learning theory |
| 10 | Sharififard et al., 2020 | Music- and game-based education | Non-digital intervention using audio tactile performance, music, and tactile models. | No | Interactive dental casts, play-dough modeling of teeth, and music-based brushing instruction | Modified PRECEDE-PROCEED oral health promotion model |
| 11 | Effendi et al., 2021 | Reminder sticker book | Non-digital intervention (sticker-based tracking system) | No | Task completion, rewards, self-monitoring, repetition | Behavioral reinforcement |
| 12 | Kang et al., 2021 | Kinect-based game | Microsoft Kinect V2 sensor, Kinect2Scratch software | Yes | Gesture-based motion capture | Task analysis-based learning and operant conditioning |
| 13 | Sharma et al., 2021 | Snakes and ladders board game | Non-digital board game combined with flash cards | No | Turn-based game, flashcard reinforcement, competition | Brain-Based Learning Theory (BFL) |
| 14 | Shruti et al., 2021 | Storytelling with hand puppets | Not applicable (Non-digital) | No | Narrative, role-playing | Not specified |
| 16 | Zolfaghari et al., 2021 | Gamified mobile health app | Android-based mobile application | Yes | Rewards, badges, score tracking, reminders, personalized notifications | Behavioral change techniques |
| 17 | Aljafari et al., 2022 | Video-game-based education | Computer-based game (school labs), Mobile version (Android) for home use | Yes | Interactive storytelling, decision-based learning, in-game rewards, progress tracking | Behavioral change theory |
| 18 | Kumar et al., 2022 | Interactive game-based visual performance | Animated video, online quiz (Kahoot) | Yes | Animated storytelling, interactive quizzes, competition, rewards, real-time feedback | Game-based learning principles |
| 19 | Kashyap et al., 2022 | Game-based oral health education | Non-digital intervention using paper-based crosswords and quizzes, PowerPoint presentations for visual support | No | Task-based learning through crossword puzzles, quiz competitions, interactive engagement | Behavioral reinforcement and cognitive learning strategies |
| 21 | Dey et al., 2023 | Augmented reality-assisted toothbrush | AR-integrated smart toothbrush, fluorescence-based lesion scoring, smartphone application | Yes | Interactive video game, real-time feedback, progress tracking, level progression | Gamification principles |
| 24 | Jagadeson et al., 2023 | Board game-based learning | Physical board game (Dental Jumanji) | No | Turn-based gameplay, task demonstration, peer interaction, question-based progression, competition | Gamification principles |
| 27 | Saraf et al., 2023 | Role-playing educational game | Not specified | No | Role-playing, storytelling, interactive tasks | Not specified |
| 28 | Shi et al., 2023 | "Dental Truth or Dare" board game | Non-digital board game modeled after Snakes and Ladders | No | Turn-based gameplay, question/answer format, task demonstrations, rewards/penalties | Game-based learning |
| 30 | Chang et al., 2024 | Gamified chatbot | LINE messaging platform, Python, MySQL, Google Cloud Platform | Yes | Interactive quizzes, level progression, virtual rewards, animated videos, reminders | Behavior Change Wheel (BCW) |
| 31 | France et al., 2024 | Smart electric toothbrush | Smart electric toothbrush with mobile app integration | Yes | Brushing timers, progress tracking, rewards, interactive games | Not specified |
| 32 | Karkoutly et al., 2024 | Dental simulation game | Mobile game: "Baby Panda Dental Care" | Yes | Role-playing as a dentist, interactive decision-making, virtual dental procedures, animated characters, real-time feedback | Behavior guidance techniques |
| 38 | Santhosh et al., 2024 | Puzzle-solving game | Non-digital jigsaw puzzles, visual aid stickers | No | Hands-on problem-solving, visual recognition, team-based play, competition | Active learning principles |
| 39 | Shirahmadi et al., 2024 | Educational games and interactive sessions | Telegram group, educational videos/animations, posters/pamphlets | Yes | Interactive learning, role-playing, quizzes, visual reinforcements, progress tracking | Health Belief Model |
| 40 | Borrelli et al., 2025 | Gamified text messaging | SMS-based mobile text messaging system | Yes | Behavioral goal shaping, progress tracking, digital badges, interactive messages, engagement incentives | Social Cognitive Theory (SCT) |

Reference:

4. Panic K, Cauberghe V, De Pelsmacker P. Promoting dental hygiene to children: comparing traditional and interactive media following threat appeals. J Health Commun. 2014;19(5):561-76. PMID: 24393019. doi: 10.1080/10810730.2013.821551.

7. Aljafari A, Rice C, Gallagher JE, Hosey MT. An oral health education video game for high caries risk children: Study protocol for a randomized controlled trial. Trials. 2015;16(1). doi: 10.1186/s13063-015-0754-6.

10. Kumar Y, Asokan S, John B, Gopalan T. Effect of Conventional and Game-based Teaching on Oral Health Status of Children: A Randomized Controlled Trial. International journal of clinical pediatric dentistry. 2015;8(2):123-6. doi: <https://dx.doi.org/10.5005/jp-journals-10005-1297>.

12. Zolfaghari M, Shirmohammadi M, Shahhosseini H, Mokhtaran M, Mohebbi SZ. Development and evaluation of a gamified smart phone mobile health application for oral health promotion in early childhood: a randomized controlled trial. BMC Oral Health. 2021;21(1):18. PMID: 33413304. doi: 10.1186/s12903-020-01374-2.

14. Malik A, Sabharwal S, Kumar A, Singh Samant P, Singh A, Kumar Pandey V. Implementation of Game-based Oral Health Education <ovid:i>vs</ovid:i> Conventional Oral Health Education on Children's Oral Health-related Knowledge and Oral Hygiene Status. International journal of clinical pediatric dentistry. 2017;10(3):257-60. doi: <https://dx.doi.org/10.5005/jp-journals-10005-1446>.

19. Chang W-J, Chang P-C, Chang Y-H. The gamification and development of a chatbot to promote oral self-care by adopting behavior change wheel for Taiwanese children. Digit Health. 2024;10:20552076241256750. PMID: 38798886. doi: 10.1177/20552076241256750.

29. Aljafari A, ElKarmi R, Nasser O, Atef Aa, Hosey MT. A Video-Game-Based Oral Health Intervention in Primary Schools-A Randomised Controlled Trial. Dentistry journal. 2022;10(5). doi: <https://dx.doi.org/10.3390/dj10050090>.

30. Kumar KRS, Deshpande AP, Ankola AV, Sankeshwari RM, Jalihal S, Hampiholi V, et al. Effectiveness of a Visual Interactive Game on Oral Hygiene Knowledge, Practices, and Clinical Parameters among Adolescents: A Randomized Controlled Trial. Children-Basel. 2022 Dec;9(12). PMID: WOS:000902292000001. doi: 10.3390/children9121828.

31. Dey S, Deshmukh S, Umamaheshwari S, Dheeraj L, Sinchan HG. Fluorescence-based Evaluation of the Efficacy of Augmented Reality-assisted Toothbrush on Oral Hygiene Practices Among 6–8 Years Old Children. Journal of Advanced Oral Research. 2023;14(2):183-9. doi: 10.1177/23202068231193772.

32. Saraf T, Hegde R, Shah P. Comparison of “My Tooth the Happiest” educational game with standard dietary counseling for preference toward non-cariogenic food items in preschool children: A Randomized control trial. Journal of Indian Society of Pedodontics and Preventive Dentistry. 2023;41(1):35-42. doi: 10.4103/jisppd.jisppd_93_23.

33. Santhosh VN, Shankkari S, Coutinho D, Ankola AV, Sankeshwari RM, Hampiholi V, et al. Effectiveness of a toothbrushing intervention utilizing puzzle-solving game assisted with visual aids among adolescents: A single-blind randomized controlled trial. Przegl Epidemiol. 2024 Dec 10;78(3):318-25. PMID: 39660713. doi: 10.32394/pe/195139.

34. Karkoutly M, Al-Halabi MN, Laflouf M, Bshara N. Effectiveness of a dental simulation game on reducing pain and anxiety during primary molars pulpotomy compared with tell-show-do technique in pediatric patients: a randomized clinical trial. BMC Oral Health. 2024;24(1). doi: 10.1186/s12903-024-04732-6.

35. Shirahmadi S, Bashirian S, Soltanian AR, Karimi-Shahanjarini A, Vahdatinia F. Effectiveness of theory-based educational interventions of promoting oral health among elementary school students. BMC Public Health. 2024 Jan 9;24(1):130. PMID: 38195494. doi: 10.1186/s12889-023-17528-0.

36. Borrelli B, Endrighi R, Heeren T, Adams WG, Gansky SA, Werntz S, et al. Parent-Targeted Oral Health Text Messaging for Underserved Children Attending Pediatric Clinics: A Randomized Clinical Trial. JAMA Netw Open. 2025 Jan 2;8(1):e2452780. PMID: 39745701. doi: 10.1001/jamanetworkopen.2024.52780.

37. Chuko C, Chao FL, Tsai HY. Design of interactive AIDS for children's teeth cleaning habits. Advances in Science, Technology and Engineering Systems. 2020;5(2):494-9. doi: 10.25046/aj050263.

38. Kang YS, Chang YJ, Howell SR. Using a kinect-based game to teach oral hygiene in four elementary students with intellectual disabilities. J Appl Res Intellect Disabil. 2021 Mar;34(2):606-14. PMID: 33258262. doi: 10.1111/jar.12828.

40. Sharma S, Saxena S, Naik SN, Bhandari R, Shukla AK, Gupta P. Comparison between Conventional, Game-based, and Self-made Storybook-based Oral Health Education on Children's Oral Hygiene Status: A Prospective Cohort Study. International journal of clinical pediatric dentistry. 2021;14(2):273-7. doi: <https://dx.doi.org/10.5005/jp-journals-10005-1811>.

41. Sharififard N, Sargeran K, Gholami M, Zayeri F. A music- and game-based oral health education for visually impaired school children; multilevel analysis of a cluster randomized controlled trial. BMC Oral Health. 2020 May 18;20(1):144. PMID: 32423446. doi: 10.1186/s12903-020-01131-5.

42. France K, Urquhart O, Ko E, Gomez J, Ryan M, Hernandez M, et al. A Pilot Study Exploring Caregivers' Experiences Related to the Use of a Smart Toothbrush by Children with Autism Spectrum Disorder. Children (Basel). 2024 Apr 11;11(4). PMID: 38671677. doi: 10.3390/children11040460.

55. Effendi MC, Hartami E, Balbeid M, Hapsari GD. Effectiveness of reminder sticker books at increasing dental health knowledge and oral hygiene. Dental Journal. 2021;54(1):5-10. doi: 10.20473/j.djmkg.v54.i1.p5-10.

Newly added

Shruti T, Govindraju HA, Sriranga J. Incorporation of Storytelling as a Method of Oral Health Education among 3-6-year-old Preschool Children. Int J Clin Pediatr Dent. 2021 May-Jun;14(3):349-352. doi: 10.5005/jp-journals-10005-1946. PMID: 34720505; PMCID: PMC8543987.

Kashyap P, Reddy L, Sinha P, Verma I, Adwani J. Effectiveness of Game-Based Oral Health Education Method on Oral Hygiene Performance of 12-Year-Old Private School Children in Lucknow City: A field trial. Journal of Indian Association of Public Health Dentistry. 2022;20:43.

Jagadeson M, Prasad V, Priyadharshini I, Prasad H, Dharshini D, Sethi M. Effect of Game Based Education in Extension of Oral Health Knowledge among 10 -12 Year Old School Children - An Interventional Study. Journal of Oral Health and Oral Epidemiology. 2024;12(4):164-9.

Shi Y, Wu WZ, Huo A, Wang HH, Lu WB, Jin XH. Effect of Conventional and "Dental Truth or Dare" Board Game on Oral Hygiene Knowledge and Oral Hygiene Status of Preschool Children. Games Health J. 2023 Apr;12(2):125-131. doi: 10.1089/g4h.2022.0059. Epub 2022 Dec 27. PMID: 36577043.
