# Supplementary material for: The Application of Gamification in Children’s Oral Health Management: Systematic Review
Source: J Med Internet Res. 2025 Nov 4;27:e75541. doi: 10.2196/75541 (PMC12627974; doi:10.2196/75541)
Supplement: Multimedia Appendix 8 [file jmir_v27i1e75541_app8.docx]

## Appendix 8: Interventions discussed in Non-Experimental Studies

| # | Reference | Study Type | Type of Gamified Intervention | Platform/Technology Used | Digital-Based Intervention | Game Elements Used | Theoretical Framework |
| --- | --- | --- | --- | --- | --- | --- | --- |
| 5 | Reynolds et al., 2019 | Observational study | Online serious game | Web-based interactive game with Adobe Flash animation, requiring a computer or laptop | Yes | Interactive cartoons, video modelling, clickable slides, coping skill instruction2 | Psychological coping models |
| 6 | Campos et al., 2019 | Observational study | Educational mobile application | Mobile app | Yes | Story and game menus5 | Not applicable |
| 9 | Fijačko et al., 2020 | Systematic review and app evaluation | Mobile apps incorporating gamification | App stores (Google Play, Apple App Store, Windows Phone Store, Amazon Appstore) | Yes | Varies across apps; at least two gamification features8 | Not applicable |
| 15 | Zaror et al., 2021 | Scoping review | Serious games for oral health education and professional training | Computers, mobile devices, consoles (Wii, Haptic, Microsoft Kinect) | Yes | Quizzes, feedback, avatars, point-based scoring, difficulty levels, rankings, rewards | Game-based learning theory and behavioural reinforcement |
| 20 | Ajay et al., 2023 | Systematic Review | Mobile health applications targeting parents/caregivers | Mobile health applications for Android and iOS; Smart toothbrush integration | Yes | Automated feedback, progress tracking, personalized recommendations, behavioural prompts, push notifications | Theory of Planned Behavior and behaviour change strategies |
| 22 | Fegan & Hutchinson, 2023 | Commentary | Mobile health applications with gamification | Smartphone applications available on Android and iOS; Smart toothbrush integration | Yes | Progress tracking, in-app rewards, brushing reminders, and interactive learning modules | Behavior changes models and the Theory of Planned Behavior |
| 23 | Gayatri et al., 2023 | Research & Development Study | Mobile application for self-examination of dental caries and oral hygiene | Mobile application developed for Android devices18; Cartoon animation characters | Yes | Self-assessment quizzes, interactive feedback, automated scoring system | Not applicable |
| 25 | Mohammadzadeh et al., 2023 | Systematic Review | Mobile health applications for children's oral health improvement | Smartphone applications for Android and iOS; Smart toothbrush integration; SMS-based reminders | Yes | Game-based storytelling, progress tracking, reminders, interactive quizzes, and multimedia educational tools | Behavioral change theories and health information technology models |
| 26 | Rizany et al., 2023 | Literature Review | Card games designed for oral health education | Non-digital card games featuring oral health-related questions and activities; Flashcards and role-playing | No | Turn-based card game mechanics, matching exercises, question-based learning, role-playing, and storytelling | Game-based learning and behavioural reinforcement |
| 29 | Widodorini & Salsabila, 2023 | Pre-experimental study | Modified Twister educational game application | Mobile application, downloadable via a provided link; Interactive digital Twister board with quiz-based engagement | Yes | Quiz-based challenges, interactive feedback, visual learning, game-based reinforcement | Not stated |
| 33 | Mendonça et al., 2024 | Descriptive Study | Serious game designed for oral health education with culturally adapted elements | Mobile game developed using Unity; Android-based application; Tablet-based gameplay | Yes | Story-driven interactive tasks, character selection, decision-making, rewards, progression through phases | Not applicable |
| 34 | Meriç, 2024 | Cross-Sectional Study | Gamified oral hygiene mobile applications for children | Turkish Google Play Store and Apple Store; Includes animations | Yes | Badges, levels, leaderboards, interactive storytelling, brushing reminders, and goal-setting features30 | Gamification and behaviour change strategies |
| 35 | Moreira et al., 2024 | Scoping Review | Various digital interventions for oral health promotion | Mobile applications for Android and iOS; Computer-based serious games; Smart toothbrushes | Yes | Badges, goal-setting, feedback, leaderboard rankings, interactive storytelling, reward systems, visual/audio feedback, progress tracking | Self-Determination Theory, Behavior Change Wheel, gamification design principles |
| 36 | Padmanabhan et al., 2024 | Narrative Review | Oral hygiene mobile applications with gamification | Mobile applications for Android and iOS; Smart toothbrush integration; Telehealth features | Yes | Brushing timers, progress tracking, rewards, interactive storytelling, goal-setting, and leaderboards | Gamification principles, behavior change models, digital health intervention strategies |
| 37 | Patil et al., 2024 | Systematic Review of RCTs | Various game-based educational methods | Digital games on tablets/mobile devices; Non-digital board games/card interventions; PowerPoint-assisted gamification | Yes (some) | Interactive storytelling, puzzles, quizzes, rewards, real-time feedback, progress tracking, and role-playing | Cognitive learning theories, game-based learning principles, and behavior reinforcement strategies |
| 41 | Peerbhay et al., 2025 | Scoping Review | Various digital interventions for oral health promotion | Mobile phones for text-message interventions; Traditional classroom-based interactive games; Video-based education | Yes (some) | Quizzes, reward-based progress tracking, interactive storytelling, and reinforcement through digital reminders | Not explicitly stated in all studies |

Reference:

11. Fijačko N, Gosak L, Cilar L, Novšak A, Creber RM, Skok P, et al. The Effects of Gamification and Oral Self-Care on Oral Hygiene in Children: Systematic Search in App Stores and Evaluation of Apps. JMIR Mhealth Uhealth. 2020;8(7):e16365. PMID: 32673235. doi: 10.2196/16365.

18. Mendonça TS, Carvalho STd, Aljafari A, Hosey MT, Costa LR. Oral Health Education for Children: Development of a Serious Game with a User-Centered Design Approach. Games Health J. 2024;13(4):268-77. PMID: 38563685. doi: 10.1089/g4h.2023.0055.

28. Campos LFXA, Cavalcante JP, Machado DP, Marçal E, Silva PGDB, Rolim JPML. Development and Evaluation of a Mobile Oral Health Application for Preschoolers. Telemedicine and e-Health. 2019;25(6):492-8. doi: 10.1089/tmj.2018.0034.

43. Zaror C, Mariño R, Atala-Acevedo C. Current State of Serious Games in Dentistry: A Scoping Review. Games Health J. 2021;10(2). PMID: 33818135. doi: 10.1089/g4h.2020.0042.

44. Mohammadzadeh N, Gholamzadeh M, Zahednamazi S, Ayyoubzadeh SM. Mobile health applications for children's oral health improvement: A systematic review. Informatics in Medicine Unlocked. 2023 2023/01/01/;37:101189. doi: <https://doi.org/10.1016/j.imu.2023.101189>.

45. Padmanabhan V, D’Souza S, Priya SP, Rehman M, El Bahra S, Tawfiq N, et al. Harnessing the Potential of Oral Hygiene Apps for Pediatric Dental Care: A Comprehensive Narrative Review. Journal of International Dental and Medical Research. 2024;17(2):860-5.

46. Patil S, Licari FW, Bhandi S, Awan KH, Di Blasio M, Isola G, et al. Effect of game-based teaching on the oral health of children: a systematic review of randomised control trials. J Clin Pediatr Dent. 2024 Jul;48(4):26-37. PMID: 39087211. doi: 10.22514/jocpd.2024.075.

47. Peerbhay F, Mash R, Khan S. Effectiveness of oral health promotion in children and adolescents through behaviour change interventions: A scoping review. PLoS One. 2025;20(1):e0316702. PMID: 39792864. doi: 10.1371/journal.pone.0316702.

48. Reynolds PA, Donaldson AN, Liossi C, Newton JT, Donaldson NK, Arias R, et al. How families prepare their children for tooth extraction under general anaesthesia: Family and clinical predictors of non-compliance with a ‘serious game’. International Journal of Paediatric Dentistry. 2019;29(2):117-28. doi: 10.1111/ipd.12450.

49. Gayatri RW, Alma LR, Ashar M, Mohd Nor NA. Smart oral health: A mobile application for dental caries and oral hygiene self-examination. Asia-Pacific Journal of Public Health. 2023;35(8):552-4. doi: <https://dx.doi.org/10.1177/10105395231204987>.

51. Meriç E. Evaluation of the quality of oral hygiene mobile apps for children using the mobile app rating scale. Int J Med Inform. 2024 Dec;192:105612. PMID: 39236585. doi: 10.1016/j.ijmedinf.2024.105612.

52. Ajay K, Azevedo LB, Haste A, Morris AJ, Giles E, Gopu BP, et al. App-based oral health promotion interventions on modifiable risk factors associated with early childhood caries: A systematic review. Frontiers in oral health. 2023;4:1125070. doi: <https://dx.doi.org/10.3389/froh.2023.1125070>.

53. Fegan H, Hutchinson R. Is the answer to reducing early childhood caries in your pocket? Evid Based Dent. 2023 Sep;24(3):134-5. PMID: 37582973. doi: 10.1038/s41432-023-00922-3.

54. Moreira R, Silveira A, Sequeira T, Durao N, Lourenco J, Cascais I, et al. Gamification and Oral Health in Children and Adolescents: Scoping Review. Interactive journal of medical research. 2024;13:e35132. doi: <https://dx.doi.org/10.2196/35132>.

56. Widodorini T, Salsabila AN. The Use of the Modified Twister Educational Game Application as Dental and Oral Health Education Media. Malaysian Journal of Medicine and Health Sciences. 2023;19:28-32. doi: 10.47836/mjmhs.19.3.5.

57. Rizany AK, Christabella J, Sulijaya B. Implementation of Card Games as Educational Media for Dental and Oral Health in Elementary School Children: A Literature Review. Journal of International Dental and Medical Research. 2023;16(3):1323-6.

New added

Amantini SNSR, Montilha AAP, Antonelli BC, Leite KTM, Rios D, Cruvinel T, Lourenço Neto N, Oliveira TM, Machado MAAM. Using Augmented Reality to Motivate Oral Hygiene Practice in Children: Protocol for the Development of a Serious Game. JMIR Res Protoc. 2020 Jan 17;9(1):e10987. doi: 10.2196/10987. PMID: 31951216; PMCID: PMC6996757.
