# Supplementary material for: The Application of Gamification in Children’s Oral Health Management: Systematic Review
Source: J Med Internet Res. 2025 Nov 4;27:e75541. doi: 10.2196/75541 (PMC12627974; doi:10.2196/75541)
Supplement: Multimedia Appendix 9 [file jmir_v27i1e75541_app9.docx]

## Appendix 9: Summary of Key Findings in Reviewed Experimental Studies

| # | Reference | Type of Gamified Intervention | Game Elements Used | Theoretical Framework | Effectiveness Results | Behavioral Impact | Engagement Metrics |
| --- | --- | --- | --- | --- | --- | --- | --- |
| 1 | Panic et al., 2014 | Educational computer game | Narrative-driven gameplay, interactive tasks, decision-making elements | Extended Parallel Processing Model (EPPM) | Higher self-reported attention for the game compared to the narrative story, but no significant difference in engagement between the game and brochure | Not specified. | Higher self-reported attention for the game compared to the narrative story. |
| 2 | Aljafari et al., 2015 | Educational video game | Not specified. | Not specified. | Pending, study is ongoing. | Not yet determined. | Completion of "secret words" task to track home engagement with the game. |
| 3 | Kumar et al., 2015 | "Connect the Dots" game combined with flash cards | Not specified. | Not specified. | Game-based teaching is more effective than conventional flash card education. | Greater sustained engagement over 3 months in the game-based teaching group. | High initial participation in both groups. |
| 4 | Malik et al., 2015 | Crossword and quiz-based learning integrated with PowerPoint presentations | Crossword puzzles, quizzes, and interactive question-based learning | Cognitive Learning Theory | Game-based oral health education is more effective than conventional methods. | Higher motivation and interest reported in the game-based education group. | High participation rate in both groups. |
| 8 | Chuko et al., 2020 | Interactive toothbrush aid | Sensory feedback, gamification | Not specified. | The interactive toothbrush aid significantly increased brushing time. | Increased engagement among young children, reducing parental supervision burden. | Not specified. |
| 10 | Sharififard et al., 2020 | Music- and game-based education combined with the ATP technique | Interactive dental casts, play-dough modeling of teeth, and music-based brushing instruction | Modified PRECEDE-PROCEED oral health promotion model | High response rate (95% at first follow-up, 99% at second follow-up). | Children initially enjoyed game- and music-based learning. | Children initially enjoyed game- and music-based learning but found ATP more feasible for regular practice. |
| 11 | Effendi et al., 2021 | Sticker chart gamification | Stickers, visual aids, rewards | Behavioral reinforcement and habit formation | Significant increase in dental health knowledge: Control group mean = 64.4, Treatment group mean = 92.5 (*p* = 0.000). Significant improvement in oral hygiene: Control group OHI-S = 1.68, Treatment group OHI-S = 0.78 (*p* = 0.000). | Significant correlation was found between sticker adherence and OHI-S reduction (*p* = 0.046). | Not specified. |
| 12 | Kang et al., 2021 | Gesture-based motion capture game | Gesture-based interaction, virtual feedback | Not specified. | Significant improvement (*p* < 0.001) in all participants, with skill retention after four weeks post-intervention. | Enhanced brushing independence observed across all participants. | Participants motivated by interactive game elements. |
| 13 | Sharma et al., 2021 | Snakes and ladders board game combined with flash cards | Board game, flash cards | Not specified. | Storybook group (Group III) showed the greatest reduction in debris score, followed by the game-based group (Group II) and the conventional group (Group I). | Not specified. | Not specified. |
| 14 | Shruti et al., 2021 | Storytelling using hand puppets | Narrative, hand puppets | Not specified. | Significant improvement in overall KAP scores: Pretest: 7.52 ± 1.95, Posttest: 8.60 ± 1.55 (*p* = 0.0001, effect size = 0.2)16. Knowledge increase: 2.97 ± 1.02 → 3.63 ± 0.78 (*p* = 0.0001, effect size = 0.3). | Not specified. | Engagement levels based on children's responses and attentiveness. |
| 16 | Zolfaghari et al., 2021 | Gamified mobile health app | Points, badges, rewards, progress tracking, virtual characters and backgrounds | Behavioral change techniques and gamification strategies | Oral health knowledge scores improved significantly in both groups, but children in the gamified app group showed superior plaque control (*p* < 0.05). | Mothers in the gamified group engaged more regularly with the app, and better plaque control was observed in children. | Regular usage rate was 48.3% for the simple app group and 72.4% for the gamified app group. |
| 17 | Aljafari et al., 2022 | Video-game-based oral health education | Interactive storytelling, decision-based learning, in-game rewards, progress tracking | Behavioral change theory and interactive digital learning | Dietary knowledge improved in the intervention group from 56.5 to 57.8 (*p* = 0.019). | Not specified. | Low home engagement, with only 27% of children downloading the game at home. |
| 18 | Kumar et al., 2022 | Interactive game-based visual performance (IGVP) technique | Kahoot quiz, visual aids | Not specified. | Reduction in plaque scores in the IGVP group by 63.4% (p < 0.001)21. Reduction in gingival scores in the IGVP group by 58.7% (p < 0.001). Knowledge scores in the IGVP group improved by 22.4%. | Not specified. | Kahoot quiz participation rate was 100%. |
| 19 | Kashyap et al., 2022 | Game-based oral health education | Not specified. | Not specified. | Oral health knowledge scores improved significantly in the game-based group. | Not specified. | Not specified. |
| 21 | Dey et al., 2023 | Augmented reality-assisted toothbrush | Interactive video game application for guiding brushing techniques | Not specified. | Significant reduction in bacterial count for both groups, with greater improvement in the augmented reality-assisted group (*p* = 0.023). | Not specified. | Not specified. |
| 24 | Jagadeson et al., 2023 | Dental Jumanji game combined with conventional lectures | Board game with dice and task-based questions | Not specified. | The effect size was 0.64 at post-test and 0.85 at follow-up, indicating a strong impact of the gamified intervention. | The game-based intervention improved knowledge retention and engagement. | Children found the Dental Jumanji game engaging and interactive. |
| 27 | Saraf et al., 2023 | "My Tooth the Happiest" educational game | Role-playing, storytelling | Not specified. | The game-based approach was more effective than standard dietary counselling in improving preschool children's preference for non-cariogenic food. | Not specified. | Not specified. |
| 28 | Shi et al., 2023 | "Dental Truth or Dare" board game | Board game, questions, rewards | Game-based learning and cognitive reinforcement strategies | Children in the board game group demonstrated significantly higher oral hygiene knowledge scores at all post-intervention time points compared to the traditional instruction group. | Not specified. | Increased attentiveness and participation among children exposed to game-based learning. |
| 30 | Chang et al., 2024 | Gamified chatbot | Quizzes, rewards, progress tracking | Behavior Change Wheel | The chatbot’s likeability score averaged 4.32 out of 5. The mean usability score (CUQ) was 79.91. | Not specified. | Mean usability score (CUQ) was 79.91, indicating good usability32. Chatbot’s likeability score averaged 4.32 out of 5, showing high user satisfaction. |
| 31 | France et al., 2024 | Smart toothbrush with gamified app | Rewards, levels, collectibles | Not specified. | 58.8% of caregivers reported their child brushed more often. 100% of children brushed at least twice daily by the end of the study. | Brushing duration improved, with an average of 1 minute and 58 seconds per session. Caregivers reported that children enjoyed brushing more and experienced less distress. | High adoption rate, with all children using the app regularly. Children enjoyed brushing more and experienced less distress. |
| 32 | Karkoutly et al., 2024 | Dental simulation game | Role-playing as a dentist, interactive decision-making, virtual dental procedures | Behavior guidance techniques and game-based learning | The game group had significantly lower pulse rates at t1 (*p* = 0.012) and t2 (*p* = 0.015) compared to the TSD group. | The game provided an interactive and engaging experience, making dental procedures feel less intimidating. | Higher participation and reduced distress compared to the TSD technique. |
| 38 | Santhosh et al., 2024 | Jigsaw Puzzle-assisted Visual Reinforcement (JPVR) technique | Jigsaw puzzles, visual aid stickers | Not specified. | At three months, the JPVR group had significantly higher knowledge (6.08 ± 1.67) and practice (5.46 ± 1.20) scores compared to the video demonstration and OHE groups. Greatest plaque score reduction in JPVR. | JPVR approach fostered active learning and behaviour reinforcement. | The gamified intervention created a competitive and enjoyable learning environment. |
| 39 | Shirahmadi et al., 2024 | Educational games and interactive learning sessions | Visual aids, Telegram group, educational videos, quizzes | Not specified. | Increased adherence to daily brushing and flossing, as well as improved oral hygiene scores. | The percentage of children brushing twice or more per day increased by 48.5% in the intervention group, and daily flossing increased by 64.2%. The percentage of students with a “good” OHI-S score improved by 44.4%. | Students actively participated in Telegram discussions and educational activities38.... The use of gamification and interactive media contributed to sustained engagement. |
| 40 | Borrelli et al., 2025 | Parent-Targeted Oral Health Text Messaging | Electronic badges, interactive quizzes, and media elements | Behavioral goal shaping, progress tracking | Increased preventive dental visits occurred in the OHT group (odds ratio: 1.51). OHT group showed significantly higher adherence to toothbrushing guidelines. | There was higher fluoride toothpaste use in the OHT group (odds ratio: 1.46). | Not specified. |

Reference:

4. Panic K, Cauberghe V, De Pelsmacker P. Promoting dental hygiene to children: comparing traditional and interactive media following threat appeals. J Health Commun. 2014;19(5):561-76. PMID: 24393019. doi: 10.1080/10810730.2013.821551.

7. Aljafari A, Rice C, Gallagher JE, Hosey MT. An oral health education video game for high caries risk children: Study protocol for a randomized controlled trial. Trials. 2015;16(1). doi: 10.1186/s13063-015-0754-6.

10. Kumar Y, Asokan S, John B, Gopalan T. Effect of Conventional and Game-based Teaching on Oral Health Status of Children: A Randomized Controlled Trial. International journal of clinical pediatric dentistry. 2015;8(2):123-6. doi: <https://dx.doi.org/10.5005/jp-journals-10005-1297>.

12. Zolfaghari M, Shirmohammadi M, Shahhosseini H, Mokhtaran M, Mohebbi SZ. Development and evaluation of a gamified smart phone mobile health application for oral health promotion in early childhood: a randomized controlled trial. BMC Oral Health. 2021;21(1):18. PMID: 33413304. doi: 10.1186/s12903-020-01374-2.

14. Malik A, Sabharwal S, Kumar A, Singh Samant P, Singh A, Kumar Pandey V. Implementation of Game-based Oral Health Education <ovid:i>vs</ovid:i> Conventional Oral Health Education on Children's Oral Health-related Knowledge and Oral Hygiene Status. International journal of clinical pediatric dentistry. 2017;10(3):257-60. doi: <https://dx.doi.org/10.5005/jp-journals-10005-1446>.

19. Chang W-J, Chang P-C, Chang Y-H. The gamification and development of a chatbot to promote oral self-care by adopting behavior change wheel for Taiwanese children. Digit Health. 2024;10:20552076241256750. PMID: 38798886. doi: 10.1177/20552076241256750.

29. Aljafari A, ElKarmi R, Nasser O, Atef Aa, Hosey MT. A Video-Game-Based Oral Health Intervention in Primary Schools-A Randomised Controlled Trial. Dentistry journal. 2022;10(5). doi: <https://dx.doi.org/10.3390/dj10050090>.

30. Kumar KRS, Deshpande AP, Ankola AV, Sankeshwari RM, Jalihal S, Hampiholi V, et al. Effectiveness of a Visual Interactive Game on Oral Hygiene Knowledge, Practices, and Clinical Parameters among Adolescents: A Randomized Controlled Trial. Children-Basel. 2022 Dec;9(12). PMID: WOS:000902292000001. doi: 10.3390/children9121828.

31. Dey S, Deshmukh S, Umamaheshwari S, Dheeraj L, Sinchan HG. Fluorescence-based Evaluation of the Efficacy of Augmented Reality-assisted Toothbrush on Oral Hygiene Practices Among 6–8 Years Old Children. Journal of Advanced Oral Research. 2023;14(2):183-9. doi: 10.1177/23202068231193772.

32. Saraf T, Hegde R, Shah P. Comparison of “My Tooth the Happiest” educational game with standard dietary counseling for preference toward non-cariogenic food items in preschool children: A Randomized control trial. Journal of Indian Society of Pedodontics and Preventive Dentistry. 2023;41(1):35-42. doi: 10.4103/jisppd.jisppd_93_23.

33. Santhosh VN, Shankkari S, Coutinho D, Ankola AV, Sankeshwari RM, Hampiholi V, et al. Effectiveness of a toothbrushing intervention utilizing puzzle-solving game assisted with visual aids among adolescents: A single-blind randomized controlled trial. Przegl Epidemiol. 2024 Dec 10;78(3):318-25. PMID: 39660713. doi: 10.32394/pe/195139.

34. Karkoutly M, Al-Halabi MN, Laflouf M, Bshara N. Effectiveness of a dental simulation game on reducing pain and anxiety during primary molars pulpotomy compared with tell-show-do technique in pediatric patients: a randomized clinical trial. BMC Oral Health. 2024;24(1). doi: 10.1186/s12903-024-04732-6.

35. Shirahmadi S, Bashirian S, Soltanian AR, Karimi-Shahanjarini A, Vahdatinia F. Effectiveness of theory-based educational interventions of promoting oral health among elementary school students. BMC Public Health. 2024 Jan 9;24(1):130. PMID: 38195494. doi: 10.1186/s12889-023-17528-0.

36. Borrelli B, Endrighi R, Heeren T, Adams WG, Gansky SA, Werntz S, et al. Parent-Targeted Oral Health Text Messaging for Underserved Children Attending Pediatric Clinics: A Randomized Clinical Trial. JAMA Netw Open. 2025 Jan 2;8(1):e2452780. PMID: 39745701. doi: 10.1001/jamanetworkopen.2024.52780.

37. Chuko C, Chao FL, Tsai HY. Design of interactive AIDS for children's teeth cleaning habits. Advances in Science, Technology and Engineering Systems. 2020;5(2):494-9. doi: 10.25046/aj050263.

38. Kang YS, Chang YJ, Howell SR. Using a kinect-based game to teach oral hygiene in four elementary students with intellectual disabilities. J Appl Res Intellect Disabil. 2021 Mar;34(2):606-14. PMID: 33258262. doi: 10.1111/jar.12828.

40. Sharma S, Saxena S, Naik SN, Bhandari R, Shukla AK, Gupta P. Comparison between Conventional, Game-based, and Self-made Storybook-based Oral Health Education on Children's Oral Hygiene Status: A Prospective Cohort Study. International journal of clinical pediatric dentistry. 2021;14(2):273-7. doi: <https://dx.doi.org/10.5005/jp-journals-10005-1811>.

41. Sharififard N, Sargeran K, Gholami M, Zayeri F. A music- and game-based oral health education for visually impaired school children; multilevel analysis of a cluster randomized controlled trial. BMC Oral Health. 2020 May 18;20(1):144. PMID: 32423446. doi: 10.1186/s12903-020-01131-5.

42. France K, Urquhart O, Ko E, Gomez J, Ryan M, Hernandez M, et al. A Pilot Study Exploring Caregivers' Experiences Related to the Use of a Smart Toothbrush by Children with Autism Spectrum Disorder. Children (Basel). 2024 Apr 11;11(4). PMID: 38671677. doi: 10.3390/children11040460.

55. Effendi MC, Hartami E, Balbeid M, Hapsari GD. Effectiveness of reminder sticker books at increasing dental health knowledge and oral hygiene. Dental Journal. 2021;54(1):5-10. doi: 10.20473/j.djmkg.v54.i1.p5-10.

Newly added

Shruti T, Govindraju HA, Sriranga J. Incorporation of Storytelling as a Method of Oral Health Education among 3-6-year-old Preschool Children. Int J Clin Pediatr Dent. 2021 May-Jun;14(3):349-352. doi: 10.5005/jp-journals-10005-1946. PMID: 34720505; PMCID: PMC8543987.

Kashyap P, Reddy L, Sinha P, Verma I, Adwani J. Effectiveness of Game-Based Oral Health Education Method on Oral Hygiene Performance of 12-Year-Old Private School Children in Lucknow City: A field trial. Journal of Indian Association of Public Health Dentistry. 2022;20:43.

Jagadeson M, Prasad V, Priyadharshini I, Prasad H, Dharshini D, Sethi M. Effect of Game Based Education in Extension of Oral Health Knowledge among 10 -12 Year Old School Children - An Interventional Study. Journal of Oral Health and Oral Epidemiology. 2024;12(4):164-9.

Shi Y, Wu WZ, Huo A, Wang HH, Lu WB, Jin XH. Effect of Conventional and "Dental Truth or Dare" Board Game on Oral Hygiene Knowledge and Oral Hygiene Status of Preschool Children. Games Health J. 2023 Apr;12(2):125-131. doi: 10.1089/g4h.2022.0059. Epub 2022 Dec 27. PMID: 36577043.
