# Supplementary material for: The Application of Gamification in Children’s Oral Health Management: Systematic Review
Source: J Med Internet Res. 2025 Nov 4;27:e75541. doi: 10.2196/75541 (PMC12627974; doi:10.2196/75541)
Supplement: Multimedia Appendix 10 [file jmir_v27i1e75541_app10.docx]

## Appendix 10: Summary of Key Findings in Reviewed Non-Experimental Studies

| # | Reference | Type of Gamified Intervention | Game Elements Used | Theoretical Framework | Effectiveness Results | Behavioral Impact | Engagement Metrics |
| --- | --- | --- | --- | --- | --- | --- | --- |
| 5 | Reynolds et al., 2019 | Online serious game | Not specified in detail. | Not applicable | Not applicable, study is observational. | Engagement in the game was correlated with families perceiving it as helpful in preparing their child. | 40% of families played the game both at home and in the hospital. Average playtime per session was 10 minutes. |
| 6 | Campos et al., 2019 | Mobile oral health application | Not specified in detail. | Not specified. | Effectiveness scores: 97.6% (Story Menu), 85.3% (Game Menu). | Not specified in detail. | Not specified in detail. |
| 7 | Amantini et al., 2020 | Augmented reality (AR)-based serious game | Motion-tracking mechanics, virtual feedback, interactive avatars, gesture-based toothbrush control, real-time error correction. | Motor learning principles and interactive learning theory. | Pending—study is in the protocol phase5. | Expected to improve brushing accuracy and adherence to proper oral hygiene practices through real-time feedback. | Gesture-based toothbrush tracking, user interaction and feedback response tracking5. |
| 9 | Fijačko et al., 2020 | Mobile apps incorporating gamification | Time pressure, virtual characters, fantasy elements, rewards. | Not specified. | The mean number of gamification features per app was 6.87 (SD 4.18) out of 316. Apps followed EBD for brushing time (94%) but lacked comprehensive oral health education. | Highlights the role of gamification in oral self-care but underscores gaps in evidence-based content. | Not specified in detail. |
| 15 | Zaror et al., 2021 | Serious games for oral health education and professional training | Quizzes, feedback, avatars, point-based scoring, difficulty levels, rankings, rewards. | Game-based learning theory and behavioral reinforcement models. | Serious games were as effective as traditional methods in improving oral health outcomes. | Serious games contributed to better knowledge retention and motivation. Some studies showed positive behavior changes in dental care routines. | High satisfaction reported with game-based learning. Motivational elements enhanced engagement. |
| 20 | Ajay et al., 2023 | Mobile health applications | Some mobile health applications incorporated gamification features. | Not specified. | Significant improvements in parental knowledge of oral health and preventive dental care were seen in two before-and-after studies. Design and usability of mobile applications play a crucial role in their effectiveness. | Mobile applications were effective in increasing parental awareness of child oral health practices. Some applications influenced dietary choices and improved brushing habits. | High parental engagement with educational content was observed in several studies. Some applications faced usability issues. |
| 22 | Fegan & Hutchinson, 2023 | Mobile health applications | Progress tracking, in-app rewards, brushing reminders, and interactive learning modules. | Behavior changes models and the Theory of Planned Behavior. | Gamified mobile application showed statistically significant improvement in plaque and gingival indices at six and twelve weeks compared to the control group. | Parents using mobile applications showed increased awareness of preventive measures. | Mobile applications incorporating gamification were more effective in engaging parents and caregivers. |
| 23 | Gayatri et al., 2023 | Mobile application for self-examination | Not specified in detail. | Not specified. | The mean OHI-S score was 0.22, suggesting good oral hygiene. | Increased awareness of dental health among children due to self-assessment capabilities. Potential for improved early detection of oral health issues. | The application was rated highly for usability, design, and educational value. Positive feedback from students on the ease of use and engaging interface. |
| 25 | Mohammadzadeh et al., 2023 | Mobile health applications | Gamification features such as points, badges, and rewards. | Not specified. | Nine studies reported statistically significant improvements in oral hygiene knowledge, plaque and gingival indices, and behavior following the use of the mobile health application. | Mobile applications with interactive components showed better retention and user satisfaction. | Usability assessments indicated that applications designed with child-friendly interfaces received higher ratings. |
| 26 | Rizany et al., 2023 | Card games for oral health education | Turn-based card game mechanics, matching exercises, question-based learning, role-playing, and storytelling. | Game-based learning and behavioral reinforcement. | All reviewed studies showed significant improvement in post-test knowledge scores compared to pre-test results. | Increased enthusiasm for learning oral health concepts through interactive play. Higher compliance with proper brushing techniques. | Children displayed greater participation and attentiveness in lessons incorporating card games. The interactive format reduced boredom and increased willingness to learn. |
| 29 | Widodorini & Salsabila, 2023 | Modified Twister educational game application | Quiz-based challenges, interactive feedback, visual learning, game-based reinforcement. | Behavior changes theory and gamification principles in education. | Not applicable, study is pre-experimental. | The intervention significantly improved children’s oral health knowledge, attitudes, and behavioral practices. Increased enthusiasm and motivation to learn about oral hygiene. | The interactive digital format encouraged higher participation. Children reported enjoying the learning experience25. |
| 33 | Mendonça et al., 2024 | Serious game for oral health education | Story-driven interactive tasks, character selection, decision-making, rewards, progression through different phases, feedback mechanisms. | User-centered design. | The global average of correct answers in the game was 75.3%, ranging from 54.5% to 90.9%. | Not applicable, study is descriptive. | Not specified in detail. |
| 34 | Meriç, 2024 | Gamified oral hygiene mobile applications | Not specified in detail. | Not specified. | Most oral hygiene apps for children achieved moderate quality scores. Apps incorporating gamification showed higher engagement, but many lacked strong evidence-based content. | Not applicable, study is app quality evaluation. | Not specified in detail. |
| 35 | Moreira et al., 2024 | Various digital interventions | Badges, goal-setting, feedback mechanisms, leaderboard rankings, interactive storytelling, reward systems, visual and audio feedback, and progress tracking. | Self-Determination Theory, Behavior Change Wheel, and gamification design principles29. | 73% of studies demonstrated positive behavior changes linked to gamification30. 80% identified goal-setting and rewards as effective mechanisms. | Increased motivation for toothbrushing and dietary changes. Reduced clinical plaque, improved gingival health, and greater adherence to oral hygiene routines. | High engagement levels observed in studies using digital rewards and leaderboards. |
| 36 | Padmanabhan et al., 2024 | Oral hygiene mobile applications | Gamification elements to promote consistent oral care routines. | Not specified. | The review highlighted the potential of mobile apps in improving oral hygiene, but also noted limitations such as the lack of long-term follow-up and potential digital equity disparities. | Mobile apps hold promise in enhancing pediatric dental care by promoting better oral hygiene practices. | Not specified in detail. |
| 41 | Harrison et al., 2024 | Various interventions | Quizzes, reward-based progress tracking, interactive storytelling, and reinforcement through digital reminders. | Not specified. | Traditional oral health education alone was insufficient for long-term behavior change. | Gamified and interactive interventions led to better retention of oral health knowledge. Interventions with reinforcement mechanisms had greater adherence. | Higher participation rates observed in interventions that included games, audiovisual materials, and interactive sessions. School-based interventions were more engaging than passive educational materials |

Reference:

11. Fijačko N, Gosak L, Cilar L, Novšak A, Creber RM, Skok P, et al. The Effects of Gamification and Oral Self-Care on Oral Hygiene in Children: Systematic Search in App Stores and Evaluation of Apps. JMIR Mhealth Uhealth. 2020;8(7):e16365. PMID: 32673235. doi: 10.2196/16365.

18. Mendonça TS, Carvalho STd, Aljafari A, Hosey MT, Costa LR. Oral Health Education for Children: Development of a Serious Game with a User-Centered Design Approach. Games Health J. 2024;13(4):268-77. PMID: 38563685. doi: 10.1089/g4h.2023.0055.

28. Campos LFXA, Cavalcante JP, Machado DP, Marçal E, Silva PGDB, Rolim JPML. Development and Evaluation of a Mobile Oral Health Application for Preschoolers. Telemedicine and e-Health. 2019;25(6):492-8. doi: 10.1089/tmj.2018.0034.

43. Zaror C, Mariño R, Atala-Acevedo C. Current State of Serious Games in Dentistry: A Scoping Review. Games Health J. 2021;10(2). PMID: 33818135. doi: 10.1089/g4h.2020.0042.

44. Mohammadzadeh N, Gholamzadeh M, Zahednamazi S, Ayyoubzadeh SM. Mobile health applications for children's oral health improvement: A systematic review. Informatics in Medicine Unlocked. 2023 2023/01/01/;37:101189. doi: <https://doi.org/10.1016/j.imu.2023.101189>.

45. Padmanabhan V, D’Souza S, Priya SP, Rehman M, El Bahra S, Tawfiq N, et al. Harnessing the Potential of Oral Hygiene Apps for Pediatric Dental Care: A Comprehensive Narrative Review. Journal of International Dental and Medical Research. 2024;17(2):860-5.

46. Patil S, Licari FW, Bhandi S, Awan KH, Di Blasio M, Isola G, et al. Effect of game-based teaching on the oral health of children: a systematic review of randomised control trials. J Clin Pediatr Dent. 2024 Jul;48(4):26-37. PMID: 39087211. doi: 10.22514/jocpd.2024.075.

47. Peerbhay F, Mash R, Khan S. Effectiveness of oral health promotion in children and adolescents through behaviour change interventions: A scoping review. PLoS One. 2025;20(1):e0316702. PMID: 39792864. doi: 10.1371/journal.pone.0316702.

48. Reynolds PA, Donaldson AN, Liossi C, Newton JT, Donaldson NK, Arias R, et al. How families prepare their children for tooth extraction under general anaesthesia: Family and clinical predictors of non-compliance with a ‘serious game’. International Journal of Paediatric Dentistry. 2019;29(2):117-28. doi: 10.1111/ipd.12450.

49. Gayatri RW, Alma LR, Ashar M, Mohd Nor NA. Smart oral health: A mobile application for dental caries and oral hygiene self-examination. Asia-Pacific Journal of Public Health. 2023;35(8):552-4. doi: <https://dx.doi.org/10.1177/10105395231204987>.

51. Meriç E. Evaluation of the quality of oral hygiene mobile apps for children using the mobile app rating scale. Int J Med Inform. 2024 Dec;192:105612. PMID: 39236585. doi: 10.1016/j.ijmedinf.2024.105612.

52. Ajay K, Azevedo LB, Haste A, Morris AJ, Giles E, Gopu BP, et al. App-based oral health promotion interventions on modifiable risk factors associated with early childhood caries: A systematic review. Frontiers in oral health. 2023;4:1125070. doi: <https://dx.doi.org/10.3389/froh.2023.1125070>.

53. Fegan H, Hutchinson R. Is the answer to reducing early childhood caries in your pocket? Evid Based Dent. 2023 Sep;24(3):134-5. PMID: 37582973. doi: 10.1038/s41432-023-00922-3.

54. Moreira R, Silveira A, Sequeira T, Durao N, Lourenco J, Cascais I, et al. Gamification and Oral Health in Children and Adolescents: Scoping Review. Interactive journal of medical research. 2024;13:e35132. doi: <https://dx.doi.org/10.2196/35132>.

56. Widodorini T, Salsabila AN. The Use of the Modified Twister Educational Game Application as Dental and Oral Health Education Media. Malaysian Journal of Medicine and Health Sciences. 2023;19:28-32. doi: 10.47836/mjmhs.19.3.5.

57. Rizany AK, Christabella J, Sulijaya B. Implementation of Card Games as Educational Media for Dental and Oral Health in Elementary School Children: A Literature Review. Journal of International Dental and Medical Research. 2023;16(3):1323-6.

New added

Amantini SNSR, Montilha AAP, Antonelli BC, Leite KTM, Rios D, Cruvinel T, Lourenço Neto N, Oliveira TM, Machado MAAM. Using Augmented Reality to Motivate Oral Hygiene Practice in Children: Protocol for the Development of a Serious Game. JMIR Res Protoc. 2020 Jan 17;9(1):e10987. doi: 10.2196/10987. PMID: 31951216; PMCID: PMC6996757.
